# Transcriptome profiling of barley in response to mineral and organic fertilizers

**DOI:** 10.1186/s12870-023-04263-2

**Published:** 2023-05-16

**Authors:** Keyvan Esmaeilzadeh-Salestani, Masoud Tohidfar, Rahele Ghanbari Moheb Seraj, Banafsheh Khaleghdoust, Indrek Keres, Hashem Marawne, Evelin Loit

**Affiliations:** 1 Chair of Crop Science and Plant Biology, Institute of Agricultural and Environmental Sciences, Estonian University of Life Sciences, Fr. R.Kreutzwaldi 1, 51014 Tartu, Estonia; 2 Department of Cell and Molecular Biology, Faculty of Life Sciences and Biotechnology, Shahid Beheshti University, Tehran, Iran; 3 Department of Horticultural Sciences, Faculty of Agriculture and Natural Resources, University of Mohaghegh Ardabili, Ardabil, Iran

**Keywords:** Barley, Organic, Conventional, Nitrogen, RNA-seq, Gene expression

## Abstract

**Background:**

Nitrogen is very important for crop yield and quality. Crop producers face the challenge of reducing the use of mineral nitrogen while maintaining food security and other ecosystem services. The first step towards understanding the metabolic responses that could be used to improve nitrogen use efficiency is to identify the genes that are up- or downregulated under treatment with different forms and rates of nitrogen. We conducted a transcriptome analysis of barley (*Hordeum vulgare* L.) cv. Anni grown in a field experiment in 2019. The objective was to compare the effects of organic (cattle manure) and mineral nitrogen (NH4NO3; 0, 40, 80 kg N ha^−1^) fertilizers on gene activity at anthesis (BBCH60) and to associate the genes that were differentially expressed between treatment groups with metabolic pathways and biological functions.

**Results:**

The highest number of differentially expressed genes (8071) was found for the treatment with the highest mineral nitrogen rate. This number was 2.6 times higher than that for the group treated with a low nitrogen rate. The lowest number (500) was for the manure treatment group. Upregulated pathways in the mineral fertilizer treatment groups included biosynthesis of amino acids and ribosomal pathways. Downregulated pathways included starch and sucrose metabolism when mineral nitrogen was supplied at lower rates and carotenoid biosynthesis and phosphatidylinositol signaling at higher mineral nitrogen rates. The organic treatment group had the highest number of downregulated genes, with phenylpropanoid biosynthesis being the most significantly enriched pathway for these genes. Genes involved in starch and sucrose metabolism and plant-pathogen interaction pathways were enriched in the organic treatment group compared with the control treatment group receiving no nitrogen input.

**Conclusion:**

These findings indicate stronger responses of genes to mineral fertilizers, probably because the slow and gradual decomposition of organic fertilizers means that less nitrogen is provided. These data contribute to our understanding of the genetic regulation of barley growth under field conditions. Identification of pathways affected by different nitrogen rates and forms under field conditions could help in the development of more sustainable cropping practices and guide breeders to create varieties with low nitrogen input requirements.

**Supplementary Information:**

The online version contains supplementary material available at 10.1186/s12870-023-04263-2.

## Background

Barley is among the most important temperate cereals, being a strategic crop that is widely used for human food and beverages as well as animal feed [1]. The world population is rapidly growing and is expected to rise to 9.6 billion by 2050. This highlights the importance of producing more foods, particularly wheat and barley, to meet the quickly increasing demand and ensure food security. The EU Sustainable Development Strategy and the Environmental Technologies Action Plan have prioritized sustainable consumption and production, including the use of environmentally friendly strategies for food production. Producing large amounts of food with minimal biodiversity loss, greenhouse gas emissions, and nutrient leaching is the main challenge for the sustainable intensification of agriculture [2].

Organic farming, which depends on adding organic fertilizers to soils, is a potential means of maintaining the organic matter and biodiversity of soil and related ecosystems [3, 4]. Although organic farming is more sustainable than conventional methods, in which mineral fertilizers and pesticides are used to increase yields, its productivity is lower [5, 6]. This yield gap raises concerns about the ability of organic farming to meet growing requirements, given the increasing world population [7]. Current breeding practices aim to decrease the yield gap between conventional and organic farming over time.

Improvement of soil quality by providing nutrients required for plant growth and development through biofertilizers is a possible solution. Nitrogen is a crucial component of key macromolecules in plants and is essential for leaf growth, stem elongation, an increase of tiller number, grain size, and yield of crops [8]. Therefore, the development of physiological and morphological traits of cereals, and subsequently their yield, depends on access to external nitrogen in agricultural soil [9]. In addition to soil amendment, modern breeding has been implemented to increase yields by exploiting genetic diversity and traits that have not yet been incorporated into elite cultivars from other cultivars and species [10], or by manipulating genes responsible for nutrient uptake and assimilation [11].

Many RNA sequencing (RNA-seq) studies have been performed to identify barley mRNAs at different scales, including genome-wide or condition/tissue/inoculation-specific studies. Quan et al. (2019) reported a difference in the transcriptome profiles of two wild barley cultivars exposed to low nitrogen conditions [12]. Transcriptome analysis showed that the two cultivars employed diverse strategies in response to heat and drought stress [13]. In other studies, candidate genes mediating salinity tolerance were identified by RNA-seq transcriptomic analysis in barley [14, 15]. The same method was used to detect genome-wide variations in barley induced by disease and gamma radiation [16, 17]. The results of those studies indicated that the responses of barley to biotic and abiotic stresses are mediated by the induction or repression of various sets of genes at specific times. However, no studies have yet demonstrated the actual impact of cropping systems on barley transcriptomic profiles.

In our pilot study, we found that barley ammonium transporters *Hv*AMT1;1 and *Hv*AMT2;1 were downregulated until heading under all treatments with added mineral fertilizers. *Hv*AMT1;1 was significantly upregulated under conventional treatments compared with organic treatments. We concluded that under field conditions, nitrogen remobilization was stimulated at the anthesis growth stage, depending on nitrogen availability in source organs [9], leading to improvements in nitrogen re-assimilation and optimization of nitrogen balance in plants [18, 19]. However, several genes are regulated by the available nutrients; investigation of changes in the barley transcriptome under different fertilization strategies is thus expected to provide insights into how fertilization management influences gene expression during barley development. Our aim was to investigate the effects of (i) mineral fertilizers, (ii) organic fertilizer (cattle manure), and (iii) pesticides on gene expression patterns under field conditions.

## Methods

### Plant materials and fertilization management

The experiment was conducted at the experimental field of the Estonian University of Life Sciences (58° 22′N, 26° 40′E) in a long-term crop rotation experiment started in 2008 and maintained since. The experimental design has been previously described in detail [9, 20]. Crop rotation includes spring barley (*Hordeum vulgare L*.) undersown with red clover, red clover (*Trifolium pratense* L.), winter wheat (*Triticum aestivum* L.), field pea (*Pisum sativum* L.), and potato (*Solanum tuberosum* L.) in that order. Certified grains of barley cv. Anni were purchased from the Estonian Crop Research Institute (www.etki.ee). The grains were sown on May 8, 2019 (500 germinating grains per m^2^, row spacing 12.5 cm) and grown using conventional and organic cropping systems. In the conventional system, the control group N0 received no fertilizer, whereas treatment groups N1 and N2 received mineral nitrogen (N) at rates of 40 and 80 kg ha^–1^ y^–1^, respectively. Mineral potassium (K) and phosphorus (P) were added to the soils of all conventional systems, except for N0, at rates of 95 and 25 kg ha^–1^y^–1^, respectively. Herbicide (MCPA, 1.0 l ha^–1^) and insecticide (Proteus, 0.75 l ha^–1^) were applied on 10.06.2019 in all conventional treatment groups. The organic cropping system included two different treatments: the control Org0, without additional fertilizers; and Org2, with cover crops and fully composted cattle manure applied at a rate of 10 t ha^–1^. Barley samples (10 plants for each biological replicate, with a total of four biological replicates for each treatment) were collected on 03.07.2019 at the anthesis growth stage (Biologische Bundesanstalt, Bundessortenamt and Chemical Industry = 60 and growing degree days (GDD) = 617.30 °C). The aboveground parts of plants were ground into powder and kept at − 80 °C until subjected to RNA-Seq analysis. The field experiment and all methods used for this study complied with relevant institutional, national and international guideline and legislation.

### Chemical analysis and quality measurement

Nitrogen contents of the leaves at the anthesis growth stage and grains after harvesting the barley were measured by the dry combustion method on a varioMAX CNS elemental analyzer (ELEMENTAR, Germany). The yield and 1000-kernel weight of samples grown under each treatment group were measured.

### Library construction and RNA sequencing

Total RNA was isolated using an RNeasy Plant Mini Kit (Qiagen, Germany) from 100 mg of ground samples. The quality of extracted RNA was assessed by 1% agarose gel electrophoresis, its purity was monitored using a NanoPhotometer® spectrophotometer (Implen, Germany), and its integrity and quantity were measured with an RNA Nano 6000 Assay Kit on a Bioanalyzer 2100 instrument (Agilent Technologies, CA, USA). One microgram of total RNA per sample (20 samples in total: five different treatments, N0, N1, N2, Org0, and Org2, with four biological replicates) was used to construct libraries with a NEBNext® UltraTM RNA Library Prep Kit for Illumina® (NEB, MA, USA) according to the manufacturer’s recommendations, using poly-T oligo-attached magnetic beads to purify the mRNA. PCR products and libraries were purified using an AMPure XP system (Beckman Coulter, Beverly, USA), and their quality was analyzed with a 2100 Bioanalyzer Instrument (Agilent, CA, USA). Finally, libraries were clustered and subsequently sequenced on an Illumina platform, NovaSeq 6000, PE150, by Novogene Co., Ltd (Cambridge, UK) to generate 150-bp paired-end reads.

### Read pre-processing and mapping to genome

The quality of raw reads (FASTQ format) was controlled and processed using fastp (version 0.22.0) [21]. Reads with poly-N or adapter sequences and low-quality reads were removed. Q20, Q30, and GC contents of the reads were obtained. Clean paired-end trimmed reads of high quality mapped to a reference genome (ftp://ftp.ensemblgenomes.org/pub/plants/release-51/fasta/hordeum_vulgare/dna/) downloaded from the Ensembl Plants database using HISAT2 (version 2.2.1) [22] with genome indexes built by hisat2-build.

### Read quantification and differential expression analysis

SAMtools (version 1.12) was used to sort mapped SAM files, and featureCounts (version 2.0.2) was used to count the number of reads mapped to each gene with sorted mapped SAM files and gene annotation files (ftp://ftp.ensemblgenomes.org/pub/plants/release-51/gff3/hordeum_vulgare) downloaded from the Ensembl Plants database as inputs [23]. Fragments per kilobase of transcript sequence per million base pairs sequence (FPKM) values were calculated for each gene, based on the gene length and count of reads aligned to the gene, to estimate gene expression levels [24]. The VennDiagram R package was used to construct venn diagram to compare the numbers of genes expressed in different groups. Differentially expressed genes (DEGs) between two groups were identified with the DESeq2 R package (version 1.28.1) [25] with a threshold of adjusted P value (*p*-adj) < 0.05, using the Benjamini–Hochberg’s method [26, 27] to control the false discovery rate (FDR). Hierarchical clustering analysis of DEGs was used to find genes with similar expression patterns among different treatment groups.

### Validation of RNA-seq analysis by real-time quantitative PCR

Total RNA was isolated using RNeasy Plant Mini Kit (Qiagen, Germany) from 100 mg of ground samples, and the quality and quantity of the extracted RNA were assessed using a NanoDrop 2000 spectrophotometer (Thermo Scientific, USA) and 1% agarose gel electrophoresis, respectively. To remove genomic DNA contamination, isolated RNA was incubated with DNase I and RiboLock RNase Inhibitor (Thermo Scientific, USA) following the manufacturer’s instructions. Two micrograms of RNA was used as a template to synthesize the first strand of cDNA using FIREScript RT cDNA Synthesis MIX with oligo (dT) and random primers (Solis BioDyne, Estonia) according to the manufacturer s’ instructions. To validate the reliability of expression patterns acquired by RNA-seq, 16 differentially up- and downregulated genes from among the DEGs were randomly selected for RT-qPCR. Specific primers for the selected genes were designed using the Oligo 7 software, version 7.60 (Molecular Biology Insights, Inc., Cascade, CO, USA) based on CDS region sequences from the EnsemblPlants database and are listed in Table 1. Gene amplification was performed using a QuantStudio™ 6 Pro Real-Time PCR System (Applied Biosystems, Germany) using 5 × HOT FIREPol® EvaGreen® qPCR Supermix (Solis BioDyne, Estonia) according to the supplier's instructions. Thermal cycling conditions comprised an initial activation for 12 min at 95 °C, followed by 45 cycles of amplification (at 95 °C for 15 s, 54 °C for 25 s, and 72 °C for 25 s). Post-amplification melting-curve analysis was undertaken by increasing the temperature from 50 °C to 95 °C to examine reaction specificity. All reactions were performed in five biological and three technical replicates. The expression value of each gene was normalized against *Hv*Act (accession number HORVU1Hr1G002840.1) and *Hv*GAPDH (accession number HORVU6Hr1G032070.1), which have been validated by Guo et al. (2020) [28] and Quan et al. (2019) [12] as reliable housekeeping genes in shoots of barley at different growth stages and under different stress conditions. The primer efficiency for each pair of primers was determined by the latest version of the LinReqPCR program (version 2017.1) by calculating the average of all individual PCR efficiencies per amplicon. Relative gene expression values were calculated by the 2^–ΔΔCT^ method according to the comparative threshold cycle [29].

**Table 1:** The primers used for validation of RNA-Seq by qRT-PCR

Accession Number	Primer Sequence (5´- 3´)	PCR Product (bp)
HORVU5Hr1G114130.1	F: CGTCCCACCATTACCACACATC	129
	R: ACACCATCGGCTTCTTCCATC	
HORVU2Hr1G119470.1	F: ACGGCAAGGGAGGAGAGC	79
	R: CTTCTCCCCCTCCCTTGCT	
HORVU6Hr1G017460.1	F: CCTCCGCCTGATCTCCATCTAT	92
	R: TCGTATGCATGCTTCCTTCGTC	
HORVU2Hr1G124020.1	F: GGGCTCGGACAAGTGGAT	110
	R: AACGGATGGCAAGAGGGG	
HORVU2Hr1G001740.1	F: AACGAGGCATGCAGTGATGG	93
	R: GGGTGTGGCGATGATGTAGC	
HORVU2Hr1G119420.1	F: GAGCGCGGCAAGGAGGAG	84
	R: CTTGGTCAGGCAGCTCTTGG	
HORVU5Hr1G056740.1	F: CCAACATCAACCCCGCCAAG	114
	R: TGTTGGGCGTGGAGAAGTGG	
HORVU7Hr1G116330.1	F: AAGGAATCATGGCCGAATGTG	114
	R: ATGGATTGTACACGCATGCTAG	
HORVU1Hr1G005460.1	F: CCGCAAATCCCCGAACAATTCC	93
	R: CGACTGTTGTTGGGGTTGGGA	
HORVU1Hr1G084510.1	F: CTCGCTCACGCCGTCACC	104
	R: CCGCTCTCACCCCACGTC	
HORVU1Hr1G000680.1	F: GGTGGGTCAATGTGTGCTCG	103
	R: GGATGGAACGGACTGCAACAC	
HORVU2Hr1G122890.1	F: CCTCGCCCATGATCTACCAGC	125
	R: CTTGGCGATGTACTTGGTGGGG	
HORVU5Hr1G056030.1	F: GTGCTGGCGCTCATCGTGG	107
	R: CCCGACGACACGAACCCAG	
HORVU1Hr1G059900.1	F: GCAGCAGAACCTCGCCGAA	78
	R: TCGCTGTAACCCTCCTGCC	
HORVU7Hr1G097730.1	F: TATGATCCCAGCGCAAACCC	102
	R: CATCTCCTGCCACAACATTTCC	
HORVU5Hr1G004510.1	F: ACCTGCAACGACACAAGAAGC	115
	R: CACGCTGGAGATGGTCTTTTGT	

### Enrichment analysis

The clusterProfiler R package (version 4.0.0) was used for gene ontology (GO) enrichment analysis of DEGs [30]. The GO terms with *p*-adj < 0.05 were considered to show a significant enriched function of DEGs. Similarly, the clusterProfiler R package was used to examine statistically enriched DEGs in Kyoto Encyclopedia of Genes and Genomes (KEGG) pathways [31].

### Protein–protein interactions (PPIs)

A PPI network of DEGs was created through blastx alignment by searching the STRING protein interaction database (http://string-db.org/) and imported into the Cytoscape (version 3.9.1) software [32] for visualization and editing.

## Results

### Chemical and quality measurement

The nitrogen content of leaves and grains of plants grown under conventional treatments N1 and N2 was higher than those in organic treatment Org2 (Table 2). Treatments N1 and N2 had significantly higher total protein content. The highest 1000-kernel weight and yield were observed in treatment N2, 45.2 g and 3.35 t ha^–1^, respectively.

**Table 2:** Nitrogen content, protein content, 1000-kernel weight and yield of barley samples under different treatments

	**Treatments**				
	**N0**	**N1**	**N2**	**Org0**	**Org2**
**Nitrogen content (leaves) (%)**	1.29 ± 0.12	1.83 ± 0.14^*^	2.30 ± 0.05^*^	1.40 ± 0.21	1.65 ± 0.13^*^
**Nitrogen content (grains) (%)**	1.79 ± 0.05	1.92 ± 0.05^*^	2.16 ± 0.05^*^	1.50 ± 0.04^*^	1.51 ± 0.01^*^
**Protein content (%)**	11.01 ± 1.24	12.59 ± 0.49^*^	14.28 ± 0.82^*^	10.57 ± 0.67	10.70 ± 1.01
**1000-kernel weight (g)**	44.19 ± 0.15	44.41 ± 0.38	45.21 ± 0.24^*^	43.81 ± 0.15	44.19 ± 0.26
**Yield (t/ha)**	1.82 ± 0.68	3.19 ± 1.59	3.35 ± 0.97^*^	2.31 ± 0.57	2.69 ± 0.72

Star represents the significant differences (*P* ≤ 0.05) between groups

### RNA-seq data quality and read mapping

In this study, a total of 938,343,621 raw reads were generated from 20 cDNA libraries through Illumina sequencing. After trimming the adaptor sequences and removing low-quality reads, a total of 919,073,637 clean reads were obtained; the complementary information of each sample is presented in File S1. An average of 84.73% clean reads were mapped to the *H. vulgare* L. reference genome using HISAT2, of which 75.68% reads were aligned to unique locations in the genome (File S2). Exon-mapped reads were the most abundant reads in all samples, accordingly for more than 90%, whereas intron- and intergenic-mapped reads represented a small percentage of the reads (Figure S1).

### Evaluation of gene expression levels

**Fig. 1:**
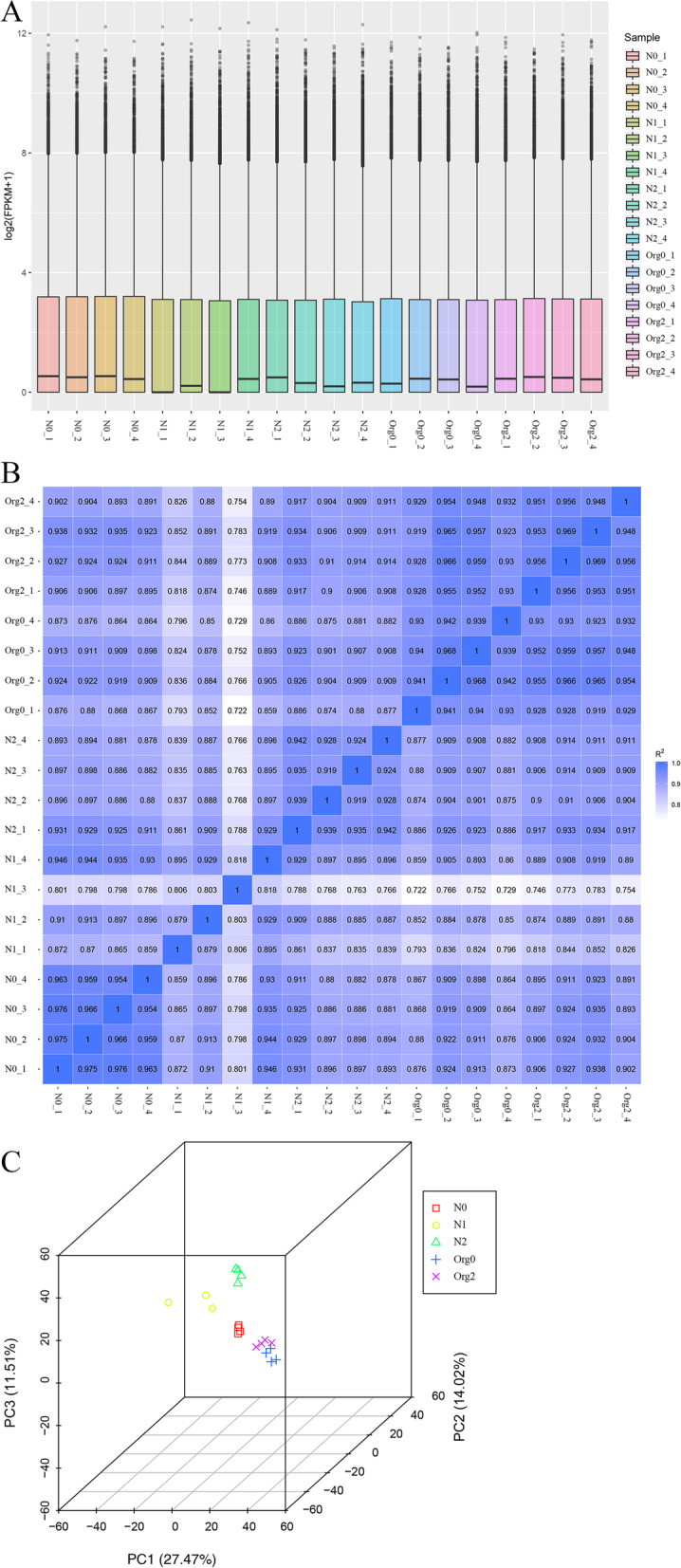
Distribution of FPKM (**A**), correlation coefficient matrix (**B**), and principal component analysis (PCA) result (**C**) of barley samples under different treatments. (note: box plots include maximum, upper quartile, mid-value, lower quartile and minimum)

**Fig. 2:**
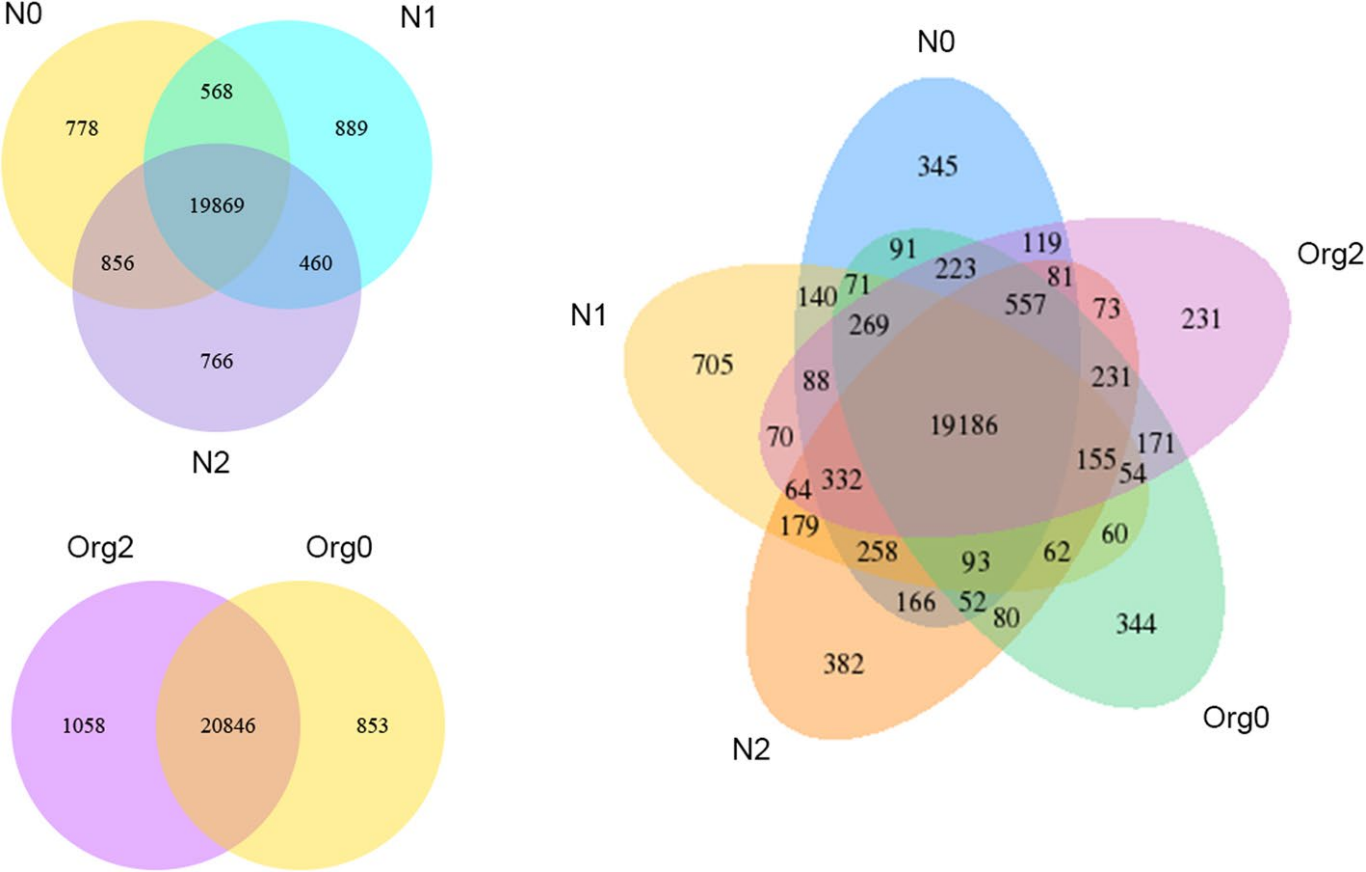
Venn diagrams of expressed genes in barley aboveground parts under different treatments

The number of reads mapped to the genome or to exons was used as a factor to represent the abundance of transcripts and was calculated using featureCounts (File S3). The FPKM, a common estimate measure of expression levels, was calculated based on both gene length and the effect of sequencing depth on read counts (File S4); FPKM > 1 was used as the threshold for the expression of a gene. When gene expression levels under different treatments were compared, overall expression levels of barley genes were found to be higher in the N1 group although similar FPKM distributions were found among treatments (Fig. 1A and File S5). Pearson’s correlation coefficients were calculated for samples, and a matrix was constructed based on the FPKM of each sample (Fig. 1B). Biological replicates had higher similarity and correlation coefficients closer to 1 (the lowest values were found for N1 biological replicates), confirming the reliability and selection of samples, as well as closer expression patterns, as confirmed by three-dimensional principal component analysis (Fig. 1C). These results demonstrate that the observed divergences were driven by the different fertilization regimes. The Venn diagram showing the numbers of genes uniquely expressed in each treatment group (Fig. 2) indicated that the expression of certain genes was induced by the different fertilization management methods. The highest and lowest numbers of uniquely expressed genes were found for N1 and Org2, respectively.

### Identification of differentially expressed genes

**Fig. 3:**
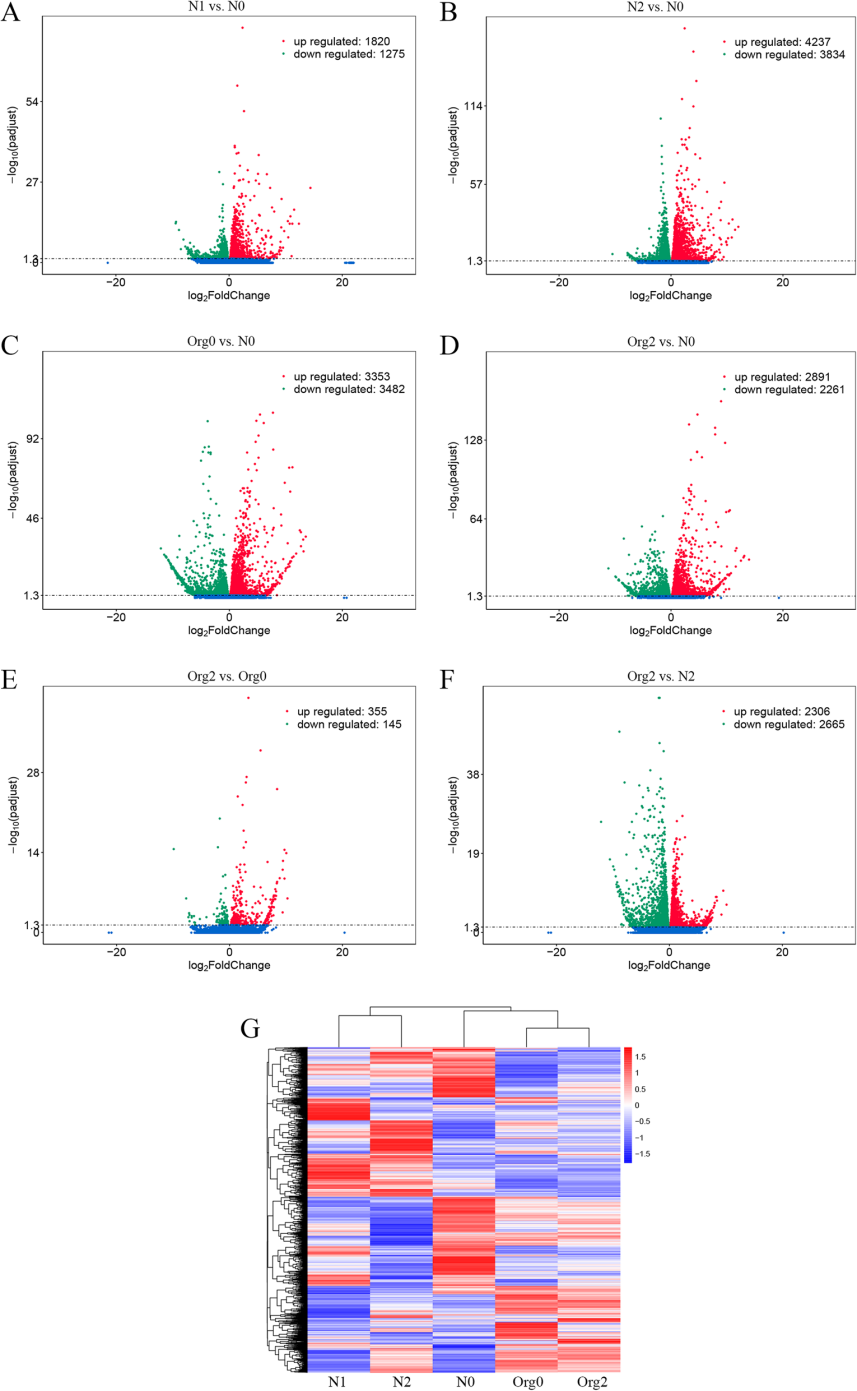
Volcano plot of barley DEGs in N1 vs. N0 (**A**), N2 vs. N0 (**B**), Org0 vs. N0 (**C**), Org2 vs. N0 (**D**), Org2 vs. Org0 (**E**), and Org2 vs. N2 (**F**). Red dots represent upregulated genes, green dots represent downregulated genes, and blue dots represent no significant difference in gene expression level. Hierarchical clustering heatmap graph using log2 (FPKM + 1) value (**G**). Red color represents genes with high expression levels; blue color represents genes with low expression levels

The read counts obtained from gene expression analysis were used to find DEGs with the DESeq2 R package. DEGs were identified in six different pairwise comparisons: N1 vs. N0, N2 vs. N0, Org0 vs. N0, Org2 vs. N0, Org2 vs. Org0, and Org2 vs. N2. The highest number of DEGs was found for N2 (8071) vs. N0, whereas N1 had fewer DEGs (3095) compared with N0 as a control. The Org2 vs. Org0 comparison yielded the lowest number of DEGs (500) (File S6 and Figure S2). More biochemical and physiological alterations took place in plants in the N2 group compared with other fertilization regimes, indicating stronger genetic regulation under this treatment. The distribution of DEGs and numbers of up- and downregulated genes of each pairwise comparison are presented in Fig. 3. N1, N2, and Org0 had more upregulated genes whereas Org2 had more downregulated genes compared with N0. Five percent of upregulated genes and 3.9% of downregulated genes were identical in four different pairwise comparisons with N0 (File S6), whereas 8.7%, 21.2%, 10.7%, and 6.4% of upregulated genes and 5.4%, 28%, 17.6%, and 5.4% of downregulated genes were unique to N1, N2, Org0, and Org2, respectively. Hierarchical clustering analysis of differential expression showed that certain clusters of genes with similar expression patterns were expressed at higher levels under specific treatment conditions (Fig. 3G), indicating involvement in the same biological activities.

### Validation of RNA-seq data

**Fig. 4:**
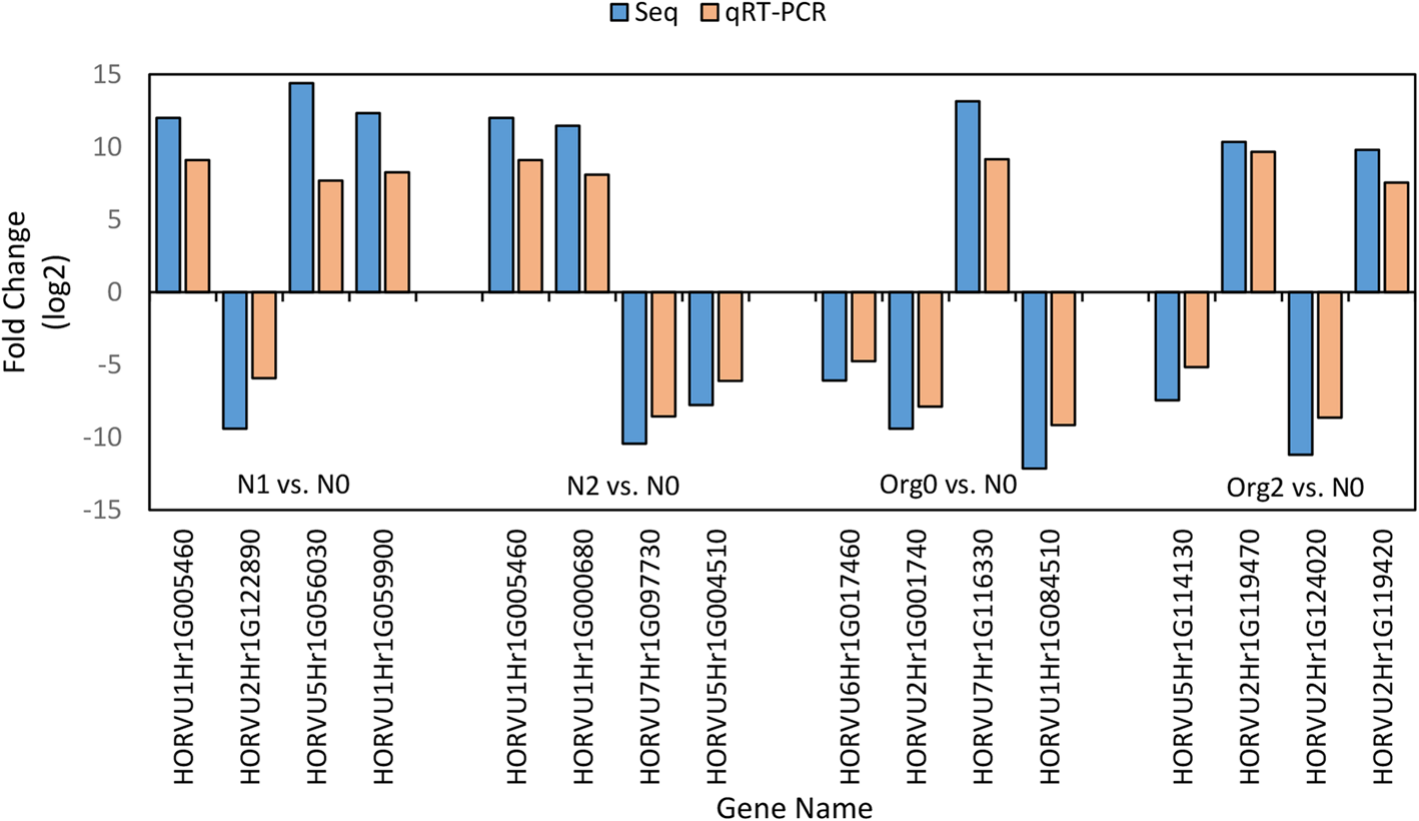
Expression validation of 16 DEGs of barley under different treatments

The qRT-PCR fold changes in the expression of 16 selected genes (four genes from each comparison) were consistent with the RNA-seq data. Linear regression analysis revealed a strong correlation between the qRT-PCR results and the sequencing data, with a correlation coefficient of 0.9837, thereby validating the sequencing procedure (Fig. 4).

### GO annotation and KEGG enrichment analysis of DEGs

**Fig. 5:**
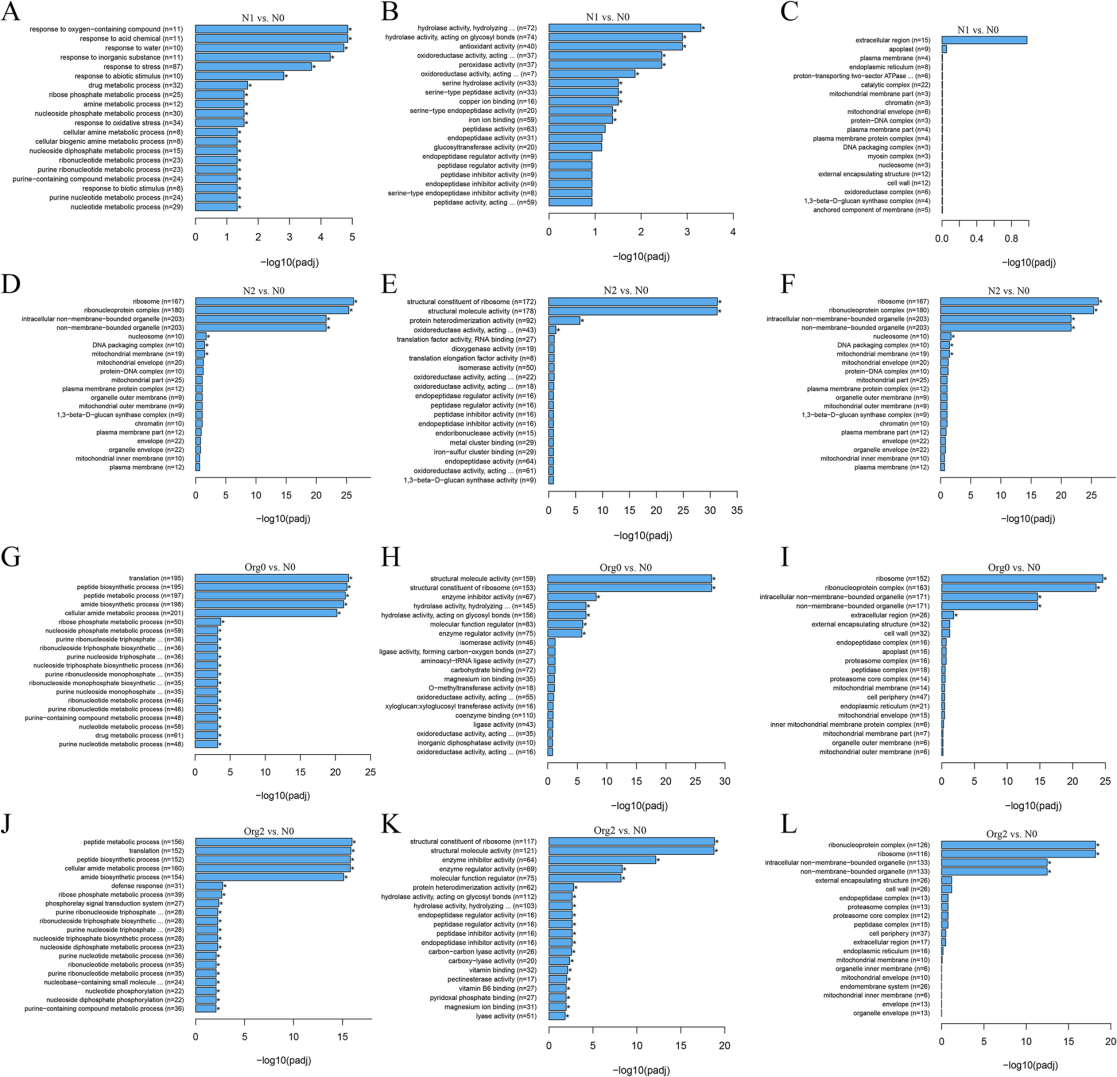
GO Enrichment Histogram of top 20 enriched terms associated with barley DEGs of N1 vs. N0 in biological processes (**A**), molecular functions (**B**), and cellular components (**C**); N2 vs. N0 in biological processes (**D**), molecular functions (**E**), and cellular components (**F**); Org0 vs. N0 in biological processes (**G**), molecular functions (**H**), and cellular components (**I**); and Org2 vs. N0 in biological processes (**G**), molecular functions (**N**), and cellular components (**L**)

Enrichment analysis was carried out to determine which biological functions or pathways were most associated with DEGs. GO analysis was performed with respect to biological processes, molecular functions, and cellular components. The GO terms associated with the DEGs are listed in File S7. In the N1 vs. N0 pairwise comparison, highly enriched GO biological process terms included stress and abiotic stimulus, as well as response to acid chemical and inorganic substances, whereas the most enriched GO molecular function terms were hydrolase, peroxidase, and antioxidant activity (Fig. 5A and B). Active cellular components were extracellular region and apoplast (Fig. 5C). In the N2 vs. N0 comparison, enriched GO included amide and peptide metabolism and chromatin and nucleosome assembly (biological processes), structural constituent of ribosome and molecule activity (molecular functions) and ribosome, nucleosome and intracellular organelles (cellular components) (Fig. 5D-F). In both the Org0 vs. N0 and Org2 vs. N0 comparisons, enriched GO terms included amide and peptide metabolism and translation process (biological processes), structural and regulatory activity (molecular functions) and ribosome, intracellular organelles, external encapsulating structure and cell wall (cellular components) (Fig. 5G-L).

**Fig. 6:**
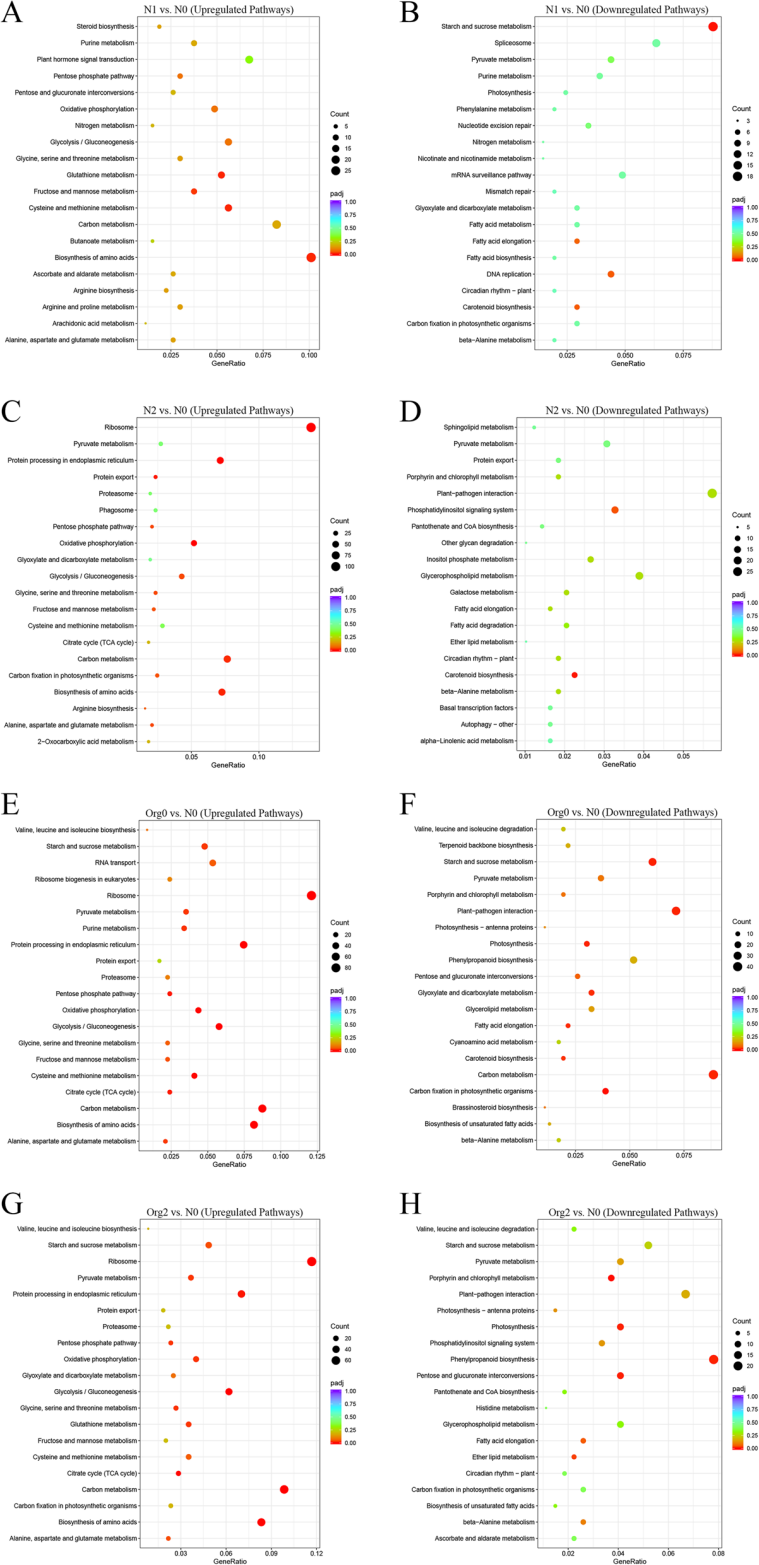
KEGG Enrichment Scatter Plot of top 20 enriched terms associated with barley DEGs. Up- (**A**) and downregulated (**B**) pathways of barley in N1 vs. N0; up- (**C**) and downregulated (**D**) pathways of barley in N2 vs. N0; up- (**E**) and downregulated (**F**) pathways of barley in Org0 vs. N0; and up- (**G**) and downregulated (**H**) pathways of barley in Org2 vs. N0. The horizontal axis is customized as GeneRatio and the vertical axis is customized as the Term's description. The size of every dot represents the number of the DEGs and the color of each dot indicates the Q-value range

KEGG analysis was carried out to annotate DEGs at the pathway level; the identified pathways are listed in File S8. In the N1 vs. N0 pairwise comparison, the fructose and mannose metabolism and biosynthesis of amino acids pathways were significantly upregulated whereas the starch and sucrose metabolism pathways showed significant downregulation (Fig. 6A and B). In the N2 vs. N0 comparison, the enriched ribosome, oxidative phosphorylation, and biosynthesis of amino acids and carbon metabolism pathways were substantially upregulated, but the carotenoid biosynthesis and phosphatidylinositol signaling system pathways were considerably downregulated (Fig. 6C and D). Among the significantly enriched pathways in both the Org0 vs. N0 and Org2 vs. N0 comparisons, ribosome, carbon metabolism, and biosynthesis of amino acids pathways were strongly upregulated. The carbon metabolism, plant-pathogen interaction, and photosynthesis pathways in the Org0 vs. N0 comparison and the porphyrin and chlorophyll metabolism, photosynthesis, and phenylpropanoid biosynthesis pathways in the Org2 vs. N0 comparison showed significant downregulation (Fig. 6E-H).

### Construction of PPI networks

**Fig. 7:**
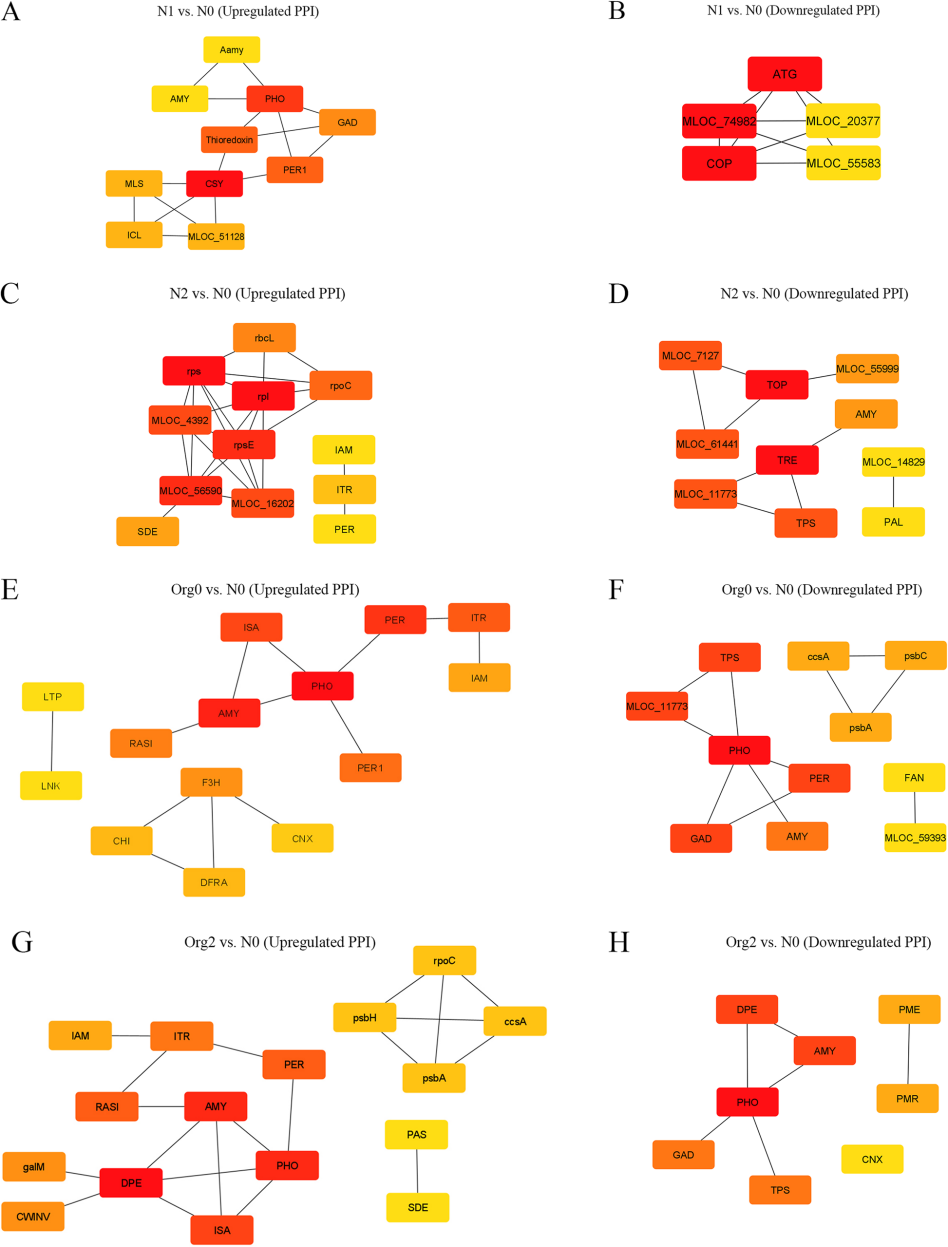
Protein–protein interaction networks. PPI network of Up- (**A**) and downregulated (**B**) DEGs of barley in N1 vs. N0; PPI network of up- (**C**) and downregulated (**D**) DEGs of barley in N2 vs. N0; PPI network of up- (**E**) and downregulated (**F**) DEGs of barley in Org0 vs. N0; and PPI network of up- (**G**) and downregulated (**H**) DEGs of barley in Org2 vs. N0. Lines indicate interactions between proteins and color intensity (from red to yellow) represents the rank of connection degree

PPI networks were constructed for up- and downregulated genes with log2 fold change ≥ 3 were constructed. In the N1 vs. N0 pairwise comparison, PHO (α-1,4 glucan phosphorylase), CSY (citrate synthase), thiroredoxin and PER1(1-Cys peroxiredoxin), which were among the upregulated proteins (Fig. 7A), and ATG (autophagy-related protein 3) and COP (Wd repeats region), which were among downregulated proteins, had the highest interactions with other portions (Fig. 7B). Upregulated proteins rpl (50S ribosomal protein L16), rps (S5 DRBM domain-containing protein) and rpsE and downregulated proteins TOP (hatpase_c domain-containing protein), TRE (trehalase) and TPS (glyco_transf_20 domain-containing protein) in N2 vs. N0 comparison had the highest interactions (Fig. 7C and D). The PHO and AMY (α-amylase) had the highest interactions with other proteins among both up- and downregulated proteins in both the Org0 vs. N0 and the Org2 vs. N0 pairwise comparisons (Fig. 7E–H).

## Discussion

### Effects of mineral fertilizers

The growth and development of plants mainly depend on the type of nutrients available in the soil and the rate at which they are available. Nitrogen, potassium, and phosphorus are the most important macronutrients for plant growth and development, and their deficiencies directly lead to reductions in crop yields [33]. We have studied the effect of different mineral nitrogen rates and organic fertilizers on crop yield and quality since 2008. Most times the mineral fertilization resulted in 24% higher total yield [20]. The barley yield was also higher in conventional treatment N2 (3.35 t ha^–1^) compared with other treatments in 2019. We have grown the barley variety Anni, which was developed 30 years ago to perform well in low- and high-nutrient systems. However, there is a need to breed new varieties that would yield well with limited nutrients and in organic conditions. Therefore, the knowledge of DEGs of plants grown under these conditions is needed. Our results showed that the combined application of NPK increased the overall expression levels of genes, and increased the number of DEGs between plants grown under N1 or N2 conditions compared with those grown under control conditions (N0).

In the low-nitrogen treatment group, N1, the DEGs were frequently involved in biological processes including response to acid chemicals and inorganic substance and response to stress, as a result of mineral nitrogen being taken up by plants. Based on our previous analysis, one reason for this could be the low soil pH (5.72) that results from long-term mineral fertilization [20]. In addition, the MAPK signaling pathway, which is activated by different stress stimuli [34], was one of the enriched pathways in treatment N1. An upregulation of abscisic acid receptor PYL9-like isoform X1 (PYR/PYL) and a downregulation of mitogen-activated protein kinase 1/2 (MPK1_2) in stress adaptation cascade of this pathway were observed (Figure S3 and Table S1). The MAPK also is involved in plant growth and development [35]. The DEGs in the N1 group were significantly involved in pathways including amino acid biosynthesis, cysteine and methionine metabolism, glutathione metabolism, fructose and mannose metabolism, and starch and sucrose metabolism. In the enriched plant hormone signal transduction pathway, an increase in expression of auxin transporter-like protein 1 (AUX1), auxin-responsive protein IAA (AUXIAA) induced cell elongation and plant growth through tryptophan metabolism (Figure S4). An upregulation of ethylene receptor (ETR) for inducing senescence in the barley hormone signal transduction pathway was shown through the cysteine and methionine metabolism. It has been reported that nitrogen supplementation affects the biosynthesis of amino acids in rice, Arabidopsis, and tea [36–38]. The enriched nitrogen metabolism pathway increased assimilatory nitrate reduction by upregulating ferredoxin-nitrite reductase (NR) and ferredoxin-nitrite reductase (NirA) genes (Figure S5). Our results showed that nitrogen content in leaves and grains was significantly higher in the N1 group than in the control group, thus indicating higher protein content (Table 2).

Furthermore, the metabolism of sulfur, which is an essential element of amino acids including methionine, cysteine, and glutathione, depends on nitrogen availability [39]. In the sulfur metabolism pathway, the expression of cysteine synthase and serine acetyltransferase 1 increased (Figure S6). The nitrogen status also influences the activity of RuBP carboxylase/oxygenase and other enzymes engaged in photosynthesis [40], leading to changes in fructose, sorbitol, sucrose, and glucose content in plants. Downregulation of genes involved in starch metabolism including glucose-1-phosphate adenylyltransferase, soluble starch synthase 2–2, and 1,4-alpha-glucan-branching enzyme 2 was shown (Figure S7). The PHO, which was among the high-interaction proteins, is involved in starch metabolism [41]. Since mitochondrial activities have to be associated with photosynthesis, regulation of the TCA cycle and CSY activity is important for plants. The CSY as one of the important enzymes involved in carbon metabolism in cooperation with enzymes engaged in nitrogen metabolism improves NUE in plants, which could be a useful strategy for decreasing the application of inorganic nitrogen fertilizers in the agricultural field [42]. The ATG, which maintains cellular homeostasis as well as protecting plant cells from nitrogen depletion or carbon starvation [43], had the highest interactions with other proteins in the network. The ATG expression was decreased because the amount of applied nitrogen added to the soil prevented deficiency in plants; this was supported by the relatively good yield (3.19 t ha^–1^).

In the medium-nitrogen treatment group, N2, where 80 kg ha^–1^ y^–1^ mineral nitrogen was applied, the number of DEGs was 2.6 times higher than that in the N1 treatment group, highlighting the impact of nitrogen on the expression patterns of genes. The nitrogen content in leaves and grains of plants grown under N2 conditions was the highest compared with all other studied treatments (2.3%). In addition, the N2 group had significantly higher 1000 kernel weight and yield compared with the control and N1 groups. The upregulation of the AUX1 and jasmonic acid-amido synthetase JAR2 isoform X1 (GH3) induced plant growth through tryptophan metabolism while the downregulation of ethylene insensitive 3-like 1 protein (EIN3) reduced the senescence through cysteine and methionine metabolism in the plant hormone signal transduction pathway (Figure S8), indicating that the plant development lasts longer to increase the yield. These DEGs were often involved in the biological processes of biosynthesis and metabolism of amides and peptides, as well as translation and assembly of nucleosomes. As higher amount of available nitrogen was present in the soil, plants expressed genes involved in the biosynthesis of amides and amino acids (peptides) that are responsible for nitrogen transport through the xylem and phloem [44]. An increased expression of NR and NirA was also observed in the nitrogen metabolism pathway in treatment N2 compared with N0 (Figure S9).

The DEGs in the N2 group were significantly involved in ribosome pathways, oxidative phosphorylation, protein processing and export, amino acid biosynthesis, and carbon metabolism. Upregulation of genes in the pathway of carbon fixation in photosynthetic organisms provides both the energy and the C-skeletons for the assimilation of ammonium during the biosynthesis of amino acids. In our previous study, the expression of *Hv*AMT1;1 increased in N2 compared to treatments N0 and N1 at the anthesis growth stage, highlighting more nitrogen is needed to be transported to the head for the grain filling. Additionally, it has been reported that the availability of nitrogen induces protein biosynthesis, ribosome biogenesis, and proteasome activity [45], consistent with a study by Yu et al. (2017) [46]. The upregulation of signal recognition particles (SPR72, SPR14, SPR19, and SPR68), signal peptidases including signal peptidase complex subunits 1, 2, and 3 (SPCS1, SPCS2, and SPCS3), and signal peptidase complex catalytic subunit SEC11A-like showed that plant is more engaged in protein biosynthesis by facilitating the protein translocation processes (Figure S10). Therefore, the plant needs more energy for driving cellular functions, which the ATP is provided by oxidative phospholylation pathway by upregulation of NADH dehydrogenases (Ndufs1, Ndufs4, Ndufs5, Ndufs6, Ndufs7, Ndufs8, Ndufv1, and Ndufv2) (Figure S11).

Two key upregulated proteins (rps and apl), which had the highest interactions, were related to ribosome pathways, indicating that increasing the nitrogen rate in the soil causes ribosomal proteins to be significantly expressed and to participate first in their own biogenesis and assembly and then to regulate protein synthesis. Barley grains grown under treatment N2 had higher protein content than those in treatment N1 (12.5 vs. 14.2%). Similarly, as part of the same field experiment, we studied another cereal, wheat, and found that the N2 group contained significantly more protein than N1 wheat (11.6 vs. 12.9%) [47]. The downregulation of TRE, one of the important proteins with high interactions, which stabilizes dehydrated proteins, enzymes, and lipid membranes as well as protecting biological structures against dehydration damage [48], showed that sufficient water (51 and 41 mm precipitation in June and July, respectively) was available for plants to grow despite having averagely high air temperate in June and July (18.6 and 16.4 °C, respectively), which led to having a good yield. It was reported that exogenous TRE enhances growth and development under low-nitrogen conditions by upregulating the metabolism of nitrogen [49], supporting that plants grown under this treatment did not suffer from low nitrogen.

### Effects of organic fertilizers

The lowest number of DEGs was observed in Org2. Similarly, Tenea et al. (2012) reported that the number of differentially expressed transcripts of wheat samples grown under organic treatment was lower than that of conventional samples when both groups were compared with the no-nitrogen sample [50]. The DEGs between the Org2 and Org0 groups were involved in the polysaccharide and glucan metabolic process and stress response (Figure S12). As the decomposition of organic fertilizers occurs gradually, less nitrogen is provided to plants compared with those treated with mineral fertilizers, leading to an increase in the polysaccharide content of plants [51]. Nitrogen metabolism pathway and particularly expression of NR and NirA, which have been upregulated under treatments with mineral fertilizers, did not change in treatment Org2 (Figure S13). Upregulated DEGs were significantly involved in the phenylpropanoid biosynthesis pathway (Figure S14). The expression of beta-glucosidase 1 and peroxidase 70-like, which are involved in the metabolism of coumarine and lignin, respectively, increased in the phenylpropanoid biosynthesis pathway (Figure S15). It has been reported that an elevated C: N ratio, resulting from either adding an external carbon input or decreasing mineral nitrogen availability, leads to an increase in phenylpropanoid metabolic activity [52, 53]. Our results showed that although the nitrogen content in leaves of plants grown under Org2 conditions was higher than those of the control and Org0 group, it was significantly less than those of plants grown under conventional treatments with low or medium amounts of mineral nitrogen. Furthermore, the nitrogen content in grains of Org2 was the same as Org0 (1.51%), which could be due to early dried leaves not allowing the translocation of nitrogen. In our previous experiment, the expression of AMTs did not alter under Org2 when compared with the control.

On the other hand, in Org2, more organic carbon is provided by cover crop residues and cattle manure, which increase microbial biomass and activity, leading to producing extracellular mucilaginous polysaccharides [54, 55]. Previously, we have shown that microbial diversity increased in organic treatments [56]. Therefore, plants could enhance the production of carbon-based secondary metabolites through the phenylpropanoid pathway. It has also been reported that organically cultivated winter wheat contains more secondary metabolites compared with plants grown under conventional treatments [57]. Therefore, polysaccharide content depends on C: N ratio. Glucans, as one of the well-known polysaccharides in nature, have different roles in the structure of the cell wall as well as they are an energetic source for metabolism [58]. Among the DEGs, endo-1,4-β-glucanases (EGs) were highly expressed. The EGs have an important role in improving the yield and quality of bakery products and animal feed [59]. The Org2 group had higher yield when compared with Org0 (2.69 vs. 2.31 t ha^–1^). In addition, our results showed that the extracellular region, cell wall, cell periphery, and apoplast were active cellular components, indicating an important regulatory role of polysaccharides in plant growth and development and in responses to environmental changes.

The PORA (dehydrogenases/reductases (SDR)) family had high interactions among the upregulated DEGs (Figure S16). The SDRs have many functions in both primary and secondary (steroids, terpenoids, alkaloids, and phenolics) metabolism [60], highlighting the role of these proteins in the phenylpropanoid biosynthesis pathway. The ABC transporter B family member 5-like (ABCB1) was upregulated under treatment Org2. The ABC transporters are involved in the uptake of nutrients, transport of secondary metabolites and hormones, regulation of stomata, responses to environmental stress, and plant and microorganism interactions [61–63]. The expression of LRR receptor-like serine/threonine-protein kinase ERECTA isoform X1 (ER/ERLs), which is involved in stomatal development increased in the MAPK signaling pathway (Figure S17). Therefore—in organic cropping systems, which rely on natural defense mechanisms—this pathway contributes to the response to biotic and abiotic stresses [64].

On the other hand, in the plant hormone signal transduction pathway (Figure S18), upregulation of auxin-responsive protein SAUR71-like (SAUR) and GH3 was observed, leading to cell enlargement and plant growth. In addition, higher expression of AUXIAA promoted ubiquitin-mediated proteolysis. It was reported that some ubiquitin-specific proteases increase grain size in rice [65]. In our experiment, the 1000-kernel weight of barley was higher in treatment Org2 compared with those in Org0. In addition, ubiquitin-mediated proteolysis allows cells to maintain the response to cellular-level signals and shifted environmental conditions [66]. The biosynthesis of cofactors was downregulated under treatment Org2. The ubiquinone biosynthesis o-methyltransferase (COQ) and lipid-binding protein involved in the biosynthesis of coenzyme Q (HMG) showed high expression in the biosynthesis pathway of ubiquinone. The COQ and HMG function in the aerobic respiratory chain, biosynthesis and metabolism of important chemical compounds, branch-chain amino acid metabolism, regulation of gene expression, and transduction of cell signals [67, 68], consistent with our results.

### Effects of pesticides

In conventional systems, in addition to mineral fertilizers, pesticides affect patterns of gene expression. To investigate the effects of pesticides, treatment Org0 was compared with N0, which received no pesticides. It has been reported that MCPA decreased the uptake of N, P, and K as well as the hormone (e.g. ABA and GA3) levels in tomatoes [69]. In our experiment, the DEGs between these two treatment groups were often involved in the biological processes of biosynthesis and metabolism of amides and peptides, and translation. As expected, defense response was one of the active biological processes in the organic treatment groups. It has been reported that plants grown under organic treatments show enhanced defense responses compared with those grown under conventional treatments [70, 71] because the gradual release of nutrients probably results in different effects on aspects of plant physiology including defense [72].

In the plant-pathogen interaction pathway (Figure S19), upregulation of PTI1-like tyrosine-protein kinase 1 (Pti1) and heat shock protein 90 kDa beta (HPS 90), as well as upregulation of respiratory burst oxidase homolog protein B-like (Rboh) were shown to induce the hypersensitive response (HR) in treatment Org0. Expression of transcription factor HBP-1b(c1)-like isoform X1 (TGA) and pathogenesis-related protein PRB1-3-like (PR-1) increased to improve diseases resistance through phenylalanine metabolism mediated by salicylic acid in the plant hormone signal transduction pathway (Figure S20). In the MAPK signaling pathway (Figure S21), the upregulation of defense genes including mitogen-activated protein kinase 2 (MPK4) and nucleoside diphosphate kinase 3 (NDPK2) induced the accumulation of reactive oxygen species, leading to cell death and defense response. The PR1 and chitinase 11 (ChiB) genes were among upregulated genes in the MAPK signaling pathway, indicating active plant defense response against the pathogens in treatment Org0, which was not treated with pesticides. In addition, the plants grown under treatment Org0 regulated the expression of genes involved in stress adaptation including PYR/PYL, probable protein phosphatase 2C 8 (PP2C), and serine/threonine-protein kinase SAPK4 (SnRK2) compared with N0.

The upregulated DEGs were involved in ribosome pathways, glycolysis/gluconeogenesis, carbon metabolism, amino acid biosynthesis, and oxidative phosphorylation. The Org0 group had higher yields and nitrogen contents in leaves compared with the N0 group. The nitrogen content of grains was low in Org0. Higher nitrogen and carbon content in the soil of treatment Org0 compared with N0 could be a reason for the enriched pathways in Org0. In addition, the pH in Org0 was closer to the ideal pH for barley growth and development compared to N0 [56]. Plant protection used in the conventional system kept the leaves green for longer, thus enabling a longer grain-filling period and more nitrogen content. High expression of PHO and AMY proteins indicated that plants in the Org0 group were engaged in the consumption of storage polysaccharides such as glycogen and starch as well as the metabolism of α-glucan. In addition, the AMY is secreted in response to pathogen attacks as a result of starch mobilization from dead cells [73, 74]. The PER, another protein with high interactions, which has roles in antioxidant defense in photosynthesis, respiration, and stress response, as well as in modulating redox signaling during development and adaptation [75], was highly expressed. These results confirm that these proteins are actively engaged in the above-mentioned upregulated pathways.

## Conclusions

This comparison between organic and conventional cropping systems showed that plants grown under organic treatments had higher activity in biological processes involving polysaccharide and glucan metabolic processes, stress, and defense response compared with conventional treatments. The DEGs of plants grown under organic treatments were significantly more engaged in pathways of organic acid metabolism, starch and sucrose metabolism compared with those in plants grown in conventional systems. On the contrary, plants grown with mineral fertilizers had higher number of DEGs, which were involved in ribosome pathways, oxidative phosphorylation, protein and amino acid biosynthesis, and carbon and nitrogen metabolism, leading to higher protein content, 1000-kernel weight and yield due to stronger responses of genes to mineral fertilizers, probably because the slow and gradual decomposition of organic fertilizers means that less nitrogen is provided. Studies such as this one, conducted under real field conditions, provide valuable information on the processes and mechanisms that are affected by different nitrogen sources and rates and can provide solutions for breeders and farmers to cope with lower nutrient input and to produce higher yields organically. Future studies will investigate the environmental effect more closely and study the DEGs on field and controlled conditions simultaneously, as well as take into analysis the samples collected from different growth stages and years.

## Supplementary Information

**Additional file 1:** **Fig. S1.** Reads distribution of each sample on genome.

**Additional file 2:** **Fig. S2.** The statistics of whole differentially expressed genes.

**Additional file 3:** **Fig. S3.** MAPK signaling pathway in N1vs N0.

**Additional file 4:** **Fig. S4.** Plant hormone signal transduction pathway in N1vs N0.

**Additional file 5:** **Fig. S5.** Nitrogen metabolism pathway in N1vs N0.

**Additional file 6:** **Fig. S6.** Sulfur metabolism pathway in N1vs N0.

**Additional file 7:** **Fig. S7.** Starch metabolism and sucrose pathway in N1vs N0.

**Additional file 8:** **Fig. S8.** Plant hormone signal transduction pathway in N2 vs N0.

**Additional file 9:** **Fig. S9.** Nitrogen metabolism pathway in N2 vs N0.

**Additional file 10:** **Fig. S10.** Protein export pathway in N2 vs N0.

**Additional file 11:** **Fig. S11.** Oxidative phospholylation pathway in N2 vs N0.

**Additional file 12:** **Fig. S12 **GO Enrichment Histogram of top 20 enriched terms associated with DEGs of Org2 vs. N2 in biological processes (BP) (A), molecular functions (MF) (B), and cellular components (CC) (C); Org2 vs. Org0 in BP (D), MF (E), and CC (F).

**Additional file 13:** **Fig. S13.** Nitrogen metabolism pathway in Org2 vs Org0.

**Additional file 14:** **Fig. S14. **KEGG Enrichment Scatter Plot of top 20 enriched terms associated with DEGs. Up- (A) and downregulated (B) pathways in Org2 vs. N2; up- (C) and downregulated (D) pathways in Org2 vs. Org0.

**Additional file 15:** **Fig. S15.** Phenylpropanoid biosynthesis pathway in Org2 vs Org0**.**

**Additional file 16:** **Fig. S16. **PPI network of Up- (A) and downregulated (B) DEGs in Org2 vs. N2; PPI network of up- (C) and downregulated (D) DEGs in Org2 vs. Org0.

**Additional file 17:** **Fig. S17.** MAPK signaling pathway in Org2 vs Org0.

**Additional file 18:** **Fig. S18.** Plant hormone signal transduction pathway in Org2 vs Org0.

**Additional file 19:** **Fig. S19.** Plant-pathogen interaction pathway in Org0 vs N0.

**Additional file 20:** **Fig. S20.** Plant hormone signal transduction pathway in Org0 vs N0.

**Additional file 21:** **Fig. S21.** MAPK signaling pathway in Org0 vs N0.

**Additional file 22:** **File S1. **Summary of data quality control. Q20 (%): percentage of bases with Phred values greater than 20. Q30(%): percentage of bases with Phred values greater than 30.

**Additional file 23:** **File S2.** Summary of mapping result of HISAT2.

**Additional file 24:** **File S3.** readcount list with the annotation of each gene.

**Additional file 25:** **File S4.** FPKM list with the annotation of each gene.

**Additional file 26:** **File S5.** statistics list of expression level for all genes.The first column is the FPKM interval, the other column is the gene number (proportion) of different samples.

**Additional file 27:** **File S6.** Gene list of all differentially expressed genes.

**Additional file 28:** **File S7.** GO enrichment list of two comparison groups.

**Additional file 29:** **File S8.** KEGG enrichment result of two comparison groups.

**Additional file 30:** **Table S1.** List of DEGs involved in the important KEGG pathways under different treatments.

## Data Availability

The datasets generated during the current study are available in the NCBI database, BioProject accession PRJNA946963 via https://www.ncbi.nlm.nih.gov/sra/PRJNA946963.

## References

[1] Zhou M, Zhang G, Li C (2009). Barley production and consumption. Genetics and improvement of barley malt quality.

[2] Schrama M, De Haan J, Kroonen M, Verstegen H, Van der Putten W (2018). Crop yield gap and stability in organic and conventional farming systems. Agric, Ecosyst Environ.

[3] Mäder P, Fliessbach A, Dubois D, Gunst L, Fried P, Niggli U (2002). Soil fertility and biodiversity in organic farming. Science.

[4] Robertson GP, Gross KL, Hamilton SK, Landis DA, Schmidt TM, Snapp SS, Swinton SM (2014). Farming for ecosystem services: An ecological approach to production agriculture. Bioscience.

[5] Tsiafouli MA, Thébault E, Sgardelis SP, De Ruiter PC, Van Der Putten WH, Birkhofer K, Hemerik L, De Vries FT, Bardgett RD, Brady MV (2015). Intensive agriculture reduces soil biodiversity across Europe. Glob Change Biol.

[6] Zhang F, Chen X, Vitousek P (2013). An experiment for the world. Nature.

[7] Trewavas A (2001). Urban myths of organic farming. Nature.

[8] Ellis RP, Marshall B (1998). Growth, yield and grain quality of barley (Hordeum vulgare L) in response to nitrogen uptake – II. Plant development and rate of germination. JXB.

[9] Esmaeilzadeh-Salestani K, Samandari_Bahraseman MR, Tohidfar M, Khaleghdoust B, Keres I, Mõttus A, Loit E. Expression of AMT1; 1 and AMT2; 1 is stimulated by mineral nitrogen and reproductive growth stage in barley under field conditions. J Plant Nutr. 2023;46(7):1246–58.

[10] Dwivedi SL, Ceccarelli S, Blair MW, Upadhyaya HD, Are AK, Ortiz R (2016). Landrace germplasm for improving yield and abiotic stress adaptation. Trends Plant Sci.

[11] Masclaux-Daubresse C, Daniel-Vedele F, Dechorgnat J, Chardon F, Gaufichon L, Suzuki A (2010). Nitrogen uptake, assimilation and remobilization in plants: challenges for sustainable and productive agriculture. Ann Bot.

[12] Quan X, Zeng J, Chen G, Zhang G (2019). Transcriptomic analysis reveals adaptive strategies to chronic low nitrogen in Tibetan wild barley. BMC Plant Biol.

[13] Cantalapiedra CP, García-Pereira MJ, Gracia MP, Igartua E, Casas AM, Contreras-Moreira B (2017). Large differences in gene expression responses to drought and heat stress between elite barley cultivar Scarlett and a Spanish landrace. Front Plant Sci.

[14] Yousefirad S, Soltanloo H, Ramezanpour SS, ZaynaliNezhad K, Shariati V (2020). The RNA-seq transcriptomic analysis reveals genes mediating salt tolerance through rapid triggering of ion transporters in a mutant barley. PLoS ONE.

[15] Zhu J, Fan Y, Li C, Shabala S, Zhao C, Hong Y, Lv C, Guo B, Xu R, Zhou M (2020). Candidate genes for salinity tolerance in barley revealed by RNA-seq analysis of near-isogenic lines. Plant Growth Regul.

[16] Ma Y, Liu M, Stiller J, Liu C (2019). A pan-transcriptome analysis shows that disease resistance genes have undergone more selection pressure during barley domestication. BMC Genomics.

[17] Tan C, Zhang X-Q, Wang Y, Wu D, Bellgard MI, Xu Y, Shu X, Zhou G, Li C (2019). Characterization of genome-wide variations induced by gamma-ray radiation in barley using RNA-Seq. BMC Genomics.

[18] Bernard SM, Habash DZ (2009). The importance of cytosolic glutamine synthetase in nitrogen assimilation and recycling. New Phytol.

[19] Miflin BJ, Habash DZ (2002). The role of glutamine synthetase and glutamate dehydrogenase in nitrogen assimilation and possibilities for improvement in the nitrogen utilization of crops. J Exp Bot.

[20] Keres I, Alaru M, Eremeev V, Talgre L, Luik A, Loit E (2020). Long-term effect of farming systems on the yield of crop rotation and soil nutrient content. Agric Food Sci.

[21] Chen S, Zhou Y, Chen Y, Gu J (2018). fastp: an ultra-fast all-in-one FASTQ preprocessor. Bioinformatics.

[22] Kim D, Paggi JM, Park C, Bennett C, Salzberg SL (2019). Graph-based genome alignment and genotyping with HISAT2 and HISAT-genotype. Nat Biotechnol.

[23] Liao Y, Smyth GK, Shi W (2014). featureCounts: an efficient general purpose program for assigning sequence reads to genomic features. Bioinformatics.

[24] Trapnell C, Williams BA, Pertea G, Mortazavi A, Kwan G, Van Baren MJ, Salzberg SL, Wold BJ, Pachter L (2010). Transcript assembly and quantification by RNA-Seq reveals unannotated transcripts and isoform switching during cell differentiation. Nat Biotechnol.

[25] Love MI, Huber W, Anders S (2014). Moderated estimation of fold change and dispersion for RNA-seq data with DESeq2. Genome Biol.

[26] Benjamini Y, Hochberg Y (1995). Controlling the false discovery rate: a practical and powerful approach to multiple testing. J Roy Stat Soc: Ser B (Methodol).

[27] Dillies M-A, Rau A, Aubert J, Hennequet-Antier C, Jeanmougin M, Servant N, Keime C, Marot G, Castel D, Estelle J (2013). A comprehensive evaluation of normalization methods for Illumina high-throughput RNA sequencing data analysis. Brief Bioinform.

[28] Guo B, Li Y, Wang S, Li D, Lv C, Xu R (2020). Characterization of the Nitrate Transporter gene family and functional identification of HvNRT2.1 in barley (Hordeum vulgare L.). PLoS One.

[29] Livak KJ, Schmittgen TD (2001). Analysis of relative gene expression data using real-time quantitative PCR and the 2(-Delta Delta C(T)) Method. Methods.

[30] Wu T, Hu E, Xu S, Chen M, Guo P, Dai Z, Feng T, Zhou L, Tang W, Zhan L (2021). clusterProfiler 4.0: A universal enrichment tool for interpreting omics data. The Innovation.

[31] Kanehisa M, Goto S (2000). KEGG: kyoto encyclopedia of genes and genomes. Nucleic Acids Res.

[32] Shannon P, Markiel A, Ozier O, Baliga NS, Wang JT, Ramage D, Amin N, Schwikowski B, Ideker T (2003). Cytoscape: a software environment for integrated models of biomolecular interaction networks. Genome Res.

[33] Ma N, Dong L, Lü W, Lü J, Meng Q, Liu P (2020). Transcriptome analysis of maize seedling roots in response to nitrogen-, phosphorus-, and potassium deficiency. Plant Soil.

[34] Danquah A, De Zélicourt A, Colcombet J, Hirt H (2014). The role of ABA and MAPK signaling pathways in plant abiotic stress responses. Biotechnol Adv.

[35] Zhang S, Klessig DF (2001). MAPK cascades in plant defense signaling. Trends Plant Sci.

[36] Balazadeh S, Schildhauer J, Araújo WL, Munné-Bosch S, Fernie AR, Proost S, Humbeck K, Mueller-Roeber B (2014). Reversal of senescence by N resupply to N-starved Arabidopsis thaliana: transcriptomic and metabolomic consequences. J Exp Bot.

[37] Chandran AKN, Jung K-H (2014). Resources for systems biology in rice. J Plant Biol.

[38] Zhang Q, Liu M, Ruan J (2017). Integrated transcriptome and metabolic analyses reveals novel insights into free amino acid metabolism in Huangjinya tea cultivar. Front Plant Sci.

[39] Reuveny Z, Dougall DK, Trinity PM (1980). Regulatory coupling of nitrate and sulfate assimilation pathways in cultured tobacco cells. Proc Natl Acad Sci.

[40] Zhang L, Sun S, Liang Y, Li B, Ma S, Wang Z, Ma B, Li M (2021). Nitrogen levels regulate sugar metabolism and transport in the shoot tips of crabapple plants. Front Plant Sci.

[41] Schupp N, Ziegler P (2004). The relation of starch phosphorylases to starch metabolism in wheat. Plant Cell Physiol.

[42] Schiavon M, Ertani A, Nardi S (2008). Effects of an alfalfa protein hydrolysate on the gene expression and activity of enzymes of the tricarboxylic acid (TCA) cycle and nitrogen metabolism in Zea mays L. J Agric Food Chem.

[43] Huo L, Guo Z, Zhang Z, Jia X, Sun Y, Sun X, Wang P, Gong X, Ma F (2020). The apple autophagy-related gene MdATG9 confers tolerance to low nitrogen in transgenic apple callus. Front Plant Sci.

[44] Ohyama T, Ohtake N, Sueyoshi K, Ono Y, Tsutsumi K, Ueno M, Tanabata S, Sato T, Takahashi Y. Amino acid metabolism and transport in soybean plants. In: Amino Acid-New Insights and Roles in Plant and Animal. Edited by Asao T, Asaduzzaman M. Croatia: InTechOpen; 2017. p. 171–96.

[45] Buszczak M, Signer RA, Morrison SJ (2014). Cellular differences in protein synthesis regulate tissue homeostasis. Cell.

[46] Yu X, Chen X, Wang L, Yang Y, Zhu X, Shao S, Cui W, Xiong F (2017). Novel insights into the effect of nitrogen on storage protein biosynthesis and protein body development in wheat caryopsis. J Exp Bot.

[47] Keres I, Alaru M, Koppel R, Altosaar I, Tosens T, Loit E (2021). The Combined Effect of Nitrogen Treatment and Weather Conditions on Wheat Protein-Starch Interaction and Dough Quality. Agriculture.

[48] Fernandez O, Béthencourt L, Quero A, Sangwan RS, Clément C (2010). Trehalose and plant stress responses: friend or foe?. Trends Plant Sci.

[49] Lin Y, Zhang J, Gao W, Chen Y, Li H, Lawlor DW, Paul MJ, Pan W (2017). Exogenous trehalose improves growth under limiting nitrogen through upregulation of nitrogen metabolism. BMC Plant Biol.

[50] Tenea GN, CordeiroRaposo F, Maquet A (2012). Comparative transcriptome profiling in winter wheat grown under different agricultural practices. J Agric Food Chem.

[51] Chung RS, Chen CC, Ng LT (2010). Nitrogen fertilization affects the growth performance, betaine and polysaccharide concentrations of Lycium barbarum. Ind Crops Prod.

[52] Fritz C, Mueller C, Matt P, Feil R, Stitt M (2006). Impact of the C-N status on the amino acid profile in tobacco source leaves. Plant, Cell Environ.

[53] Nunes-Nesi A, Fernie AR, Stitt M (2010). Metabolic and signaling aspects underpinning the regulation of plant carbon nitrogen interactions. Mol Plant.

[54] Lynch J, Bragg E, Stewart BA (1985). Microorganisms and soil aggregate stability. Advances in soil science.

[55] Roberson EB, Shennan C, Firestone MK, Sarig S (1995). Nutritional management of microbial polysaccharide production and aggregation in an agricultural soil. Soil Sci Soc Am J.

[56] Esmaeilzadeh-Salestani K, Bahram M, Seraj RGM, Gohar D, Tohidfar M, Eremeev V, Talgre L, Khaleghdoust B, Mirmajlessi SM, Luik A (2021). Cropping systems with higher organic carbon promote soil microbial diversity. Agric, Ecosyst Environ.

[57] Żuchowski J, Kapusta I, Szajwaj B, Jończyk K, Oleszek W (2009). Phenolic acid content of organic and conventionally grown winter wheat. Cereal Res Commun.

[58] Synytsya A, Novak M (2014). Structural analysis of glucans. Ann Trans Med.

[59] Annamalai N, Rajeswari MV, Balasubramanian T. Endo-1, 4-β-glucanases: role, applications and recent developments. In: Microbial enzymes in bioconversions of biomass. Edited by Gupta VK. Switzerland: Springer Cham; 2016. p. 37–45.

[60] Tonfack LB, Moummou H, Latché A, Youmbi E, Benichou M, Pech JC, Van Der Rest B (2011). The plant SDR superfamily: involvement in primary and secondary metabolism. Curr Top Plant Biol.

[61] Gräfe K, Schmitt L (2021). The ABC transporter G subfamily in Arabidopsis thaliana. J Exp Bot.

[62] Lane TS, Rempe CS, Davitt J, Staton ME, Peng Y, Soltis DE, Melkonian M, Deyholos M, Leebens-Mack JH, Chase M (2016). Diversity of ABC transporter genes across the plant kingdom and their potential utility in biotechnology. BMC Biotechnol.

[63] Yazaki K (2006). ABC transporters involved in the transport of plant secondary metabolites. FEBS Lett.

[64] Dong NQ, Lin HX (2021). Contribution of phenylpropanoid metabolism to plant development and plant–environment interactions. J Integr Plant Biol.

[65] Linden KJ, Callis J (2020). The ubiquitin system affects agronomic plant traits. J Biol Chem.

[66] Sharma B, Joshi D, Yadav PK, Gupta AK, Bhatt TK (2016). Role of Ubiquitin-Mediated Degradation System in Plant Biology. Front Plant Sci.

[67] Araújo WL, Ishizaki K, Nunes-Nesi A, Larson TR, Tohge T, Krahnert I, Witt S, Obata T, Schauer N, Graham IA (2010). Identification of the 2-hydroxyglutarate and isovaleryl-CoA dehydrogenases as alternative electron donors linking lysine catabolism to the electron transport chain of Arabidopsis mitochondria. Plant Cell.

[68] Liu M, Lu S (1898). Plastoquinone and ubiquinone in plants: biosynthesis, physiological function and metabolic engineering. Front Plant Sci.

[69] Zhang J, Guo T, Xiao Q, Wang P, Tian H (2021). Effect of 4-chloro-2-methylphenoxy acetic acid on tomato gene expression and rhizosphere bacterial communities under inoculation with phosphate-solubilizing bacteria. J Hazard Mater.

[70] Bais HP, Park S-W, Weir TL, Callaway RM, Vivanco JM (2004). How plants communicate using the underground information superhighway. Trends Plant Sci.

[71] Goh C-H, VelizVallejos DF, Nicotra AB, Mathesius U (2013). The impact of beneficial plant-associated microbes on plant phenotypic plasticity. J Chem Ecol.

[72] Hsu Y-T, Shen T-C, Hwang S-Y (2009). Soil fertility management and pest responses: a comparison of organic and synthetic fertilization. J Econ Entomol.

[73] Heitz T, Geoffroy P, Fritig B, Legrand M (1991). Two apoplastic α-amylases are induced in tobacco by virus infection. Plant Physiol.

[74] Stanley D, Farnden KJ, MacRae EA (2005). Plant α-amylases: functions and roles in carbohydrate metabolism. Biologia.

[75] Dietz K-J (2003). Plant peroxiredoxins. Annu Rev Plant Biol.

