# Supplementary material for: Transcriptome profiling of barley in response to mineral and organic fertilizers
Source: BMC Plant Biol. 2023 May 16;23:261. doi: 10.1186/s12870-023-04263-2 (PMC10186687; doi:10.1186/s12870-023-04263-2)
Supplement: Supplementary file 1 — Additional file 1: Fig. S1. Reads distribution of each sample on genome. [file 12870_2023_4263_MOESM1_ESM.zip › Figure S1 caption.docx]

**Fig. S1** Reads distribution of each sample on genome.
