# Supplementary figures and images for: Transcriptome profiling of barley in response to mineral and organic fertilizers
Source: BMC Plant Biol. 2023 May 16;23:261. doi: 10.1186/s12870-023-04263-2 (PMC10186687; doi:10.1186/s12870-023-04263-2)

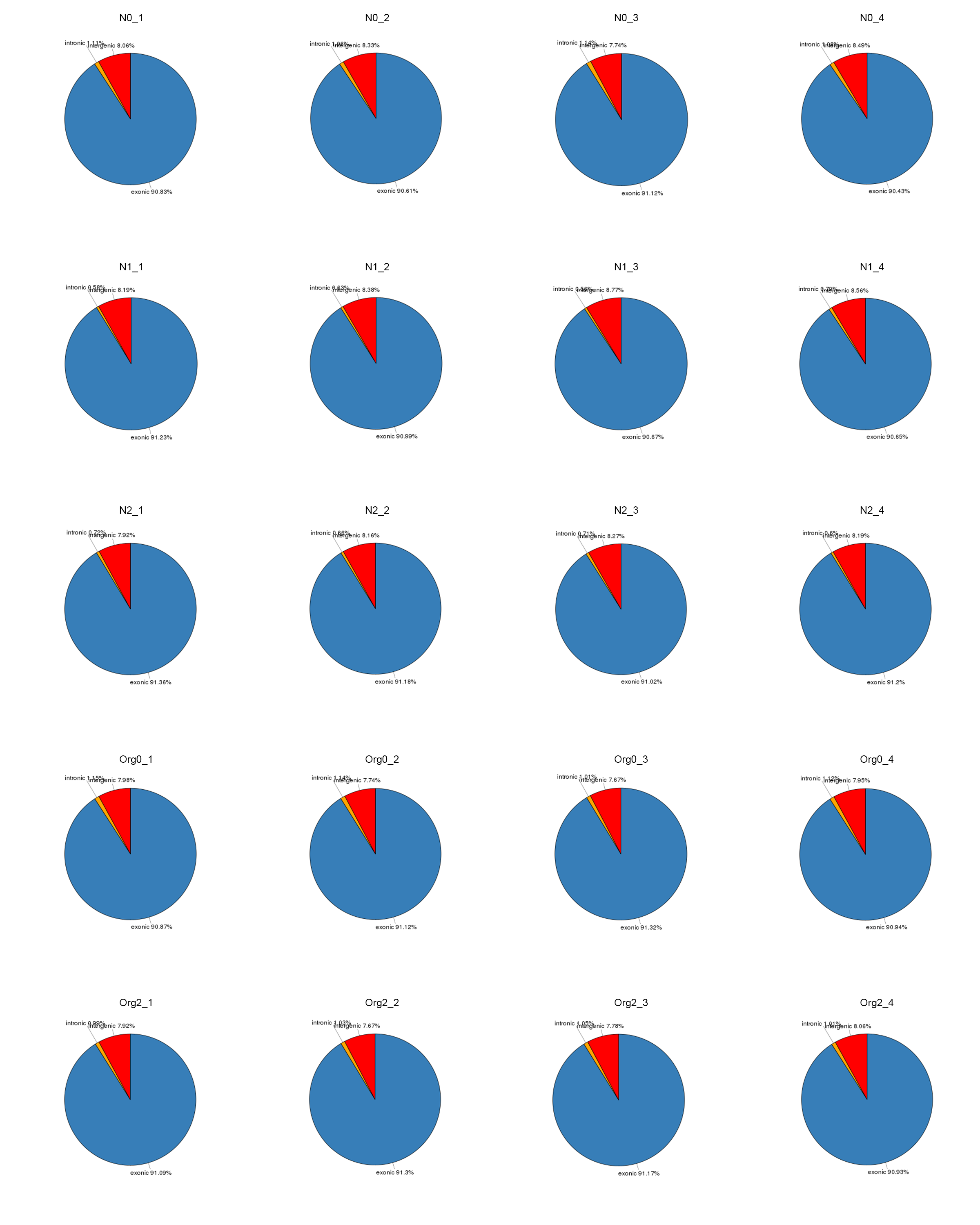

Supplement: Supplementary file 1 — Additional file 1: Fig. S1. Reads distribution of each sample on genome. [file 12870_2023_4263_MOESM1_ESM.zip › Figure S1.tif]

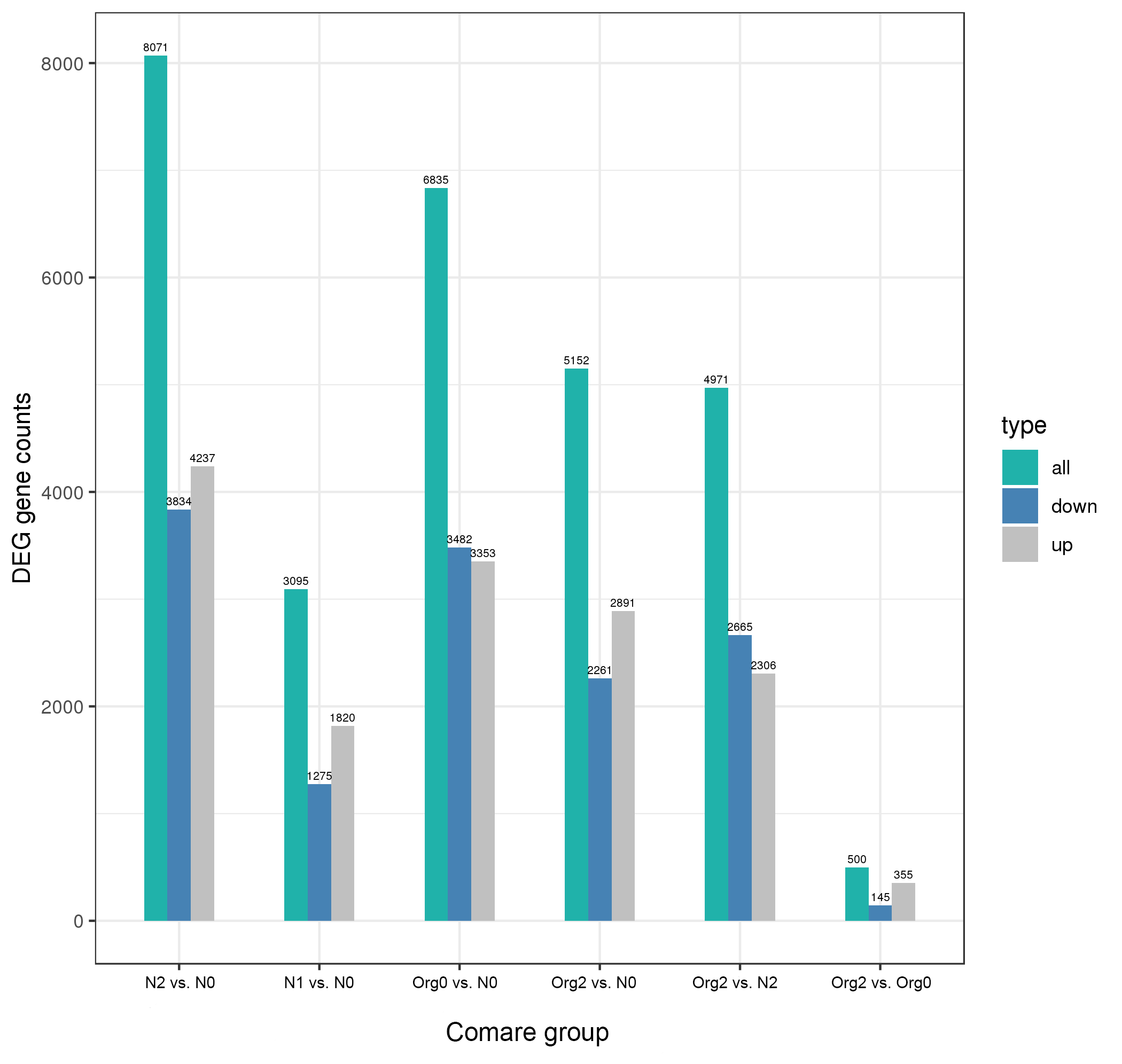

Supplement: Supplementary file 2 — Additional file 2: Fig. S2. The statistics of whole differentially expressed genes. [file 12870_2023_4263_MOESM2_ESM.zip › Figure S2.tif]

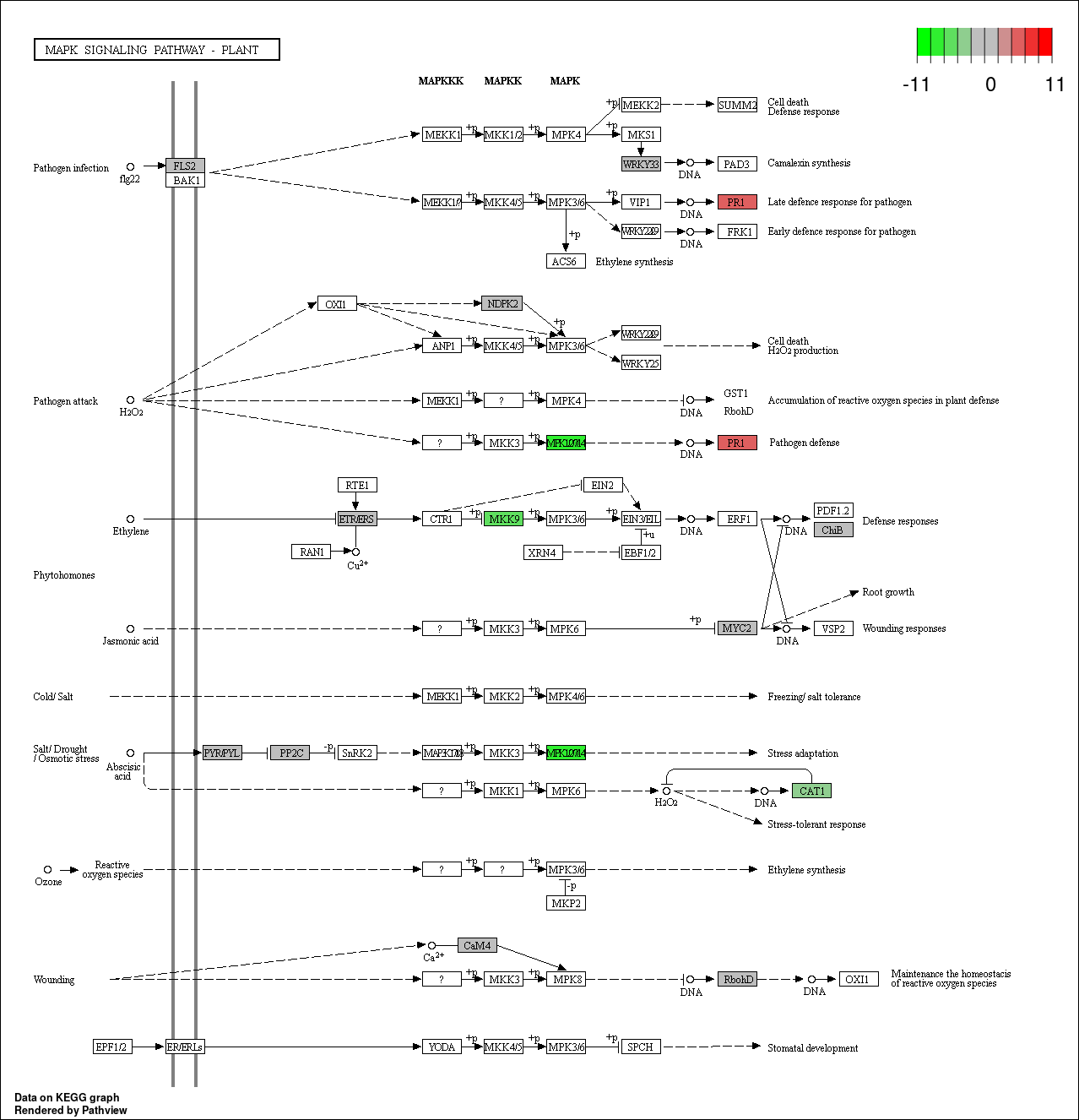

Supplement: Supplementary file 3 — Additional file 3: Fig. S3. MAPK signaling pathway in N1vs N0. [file 12870_2023_4263_MOESM3_ESM.zip › Figure S3.png]

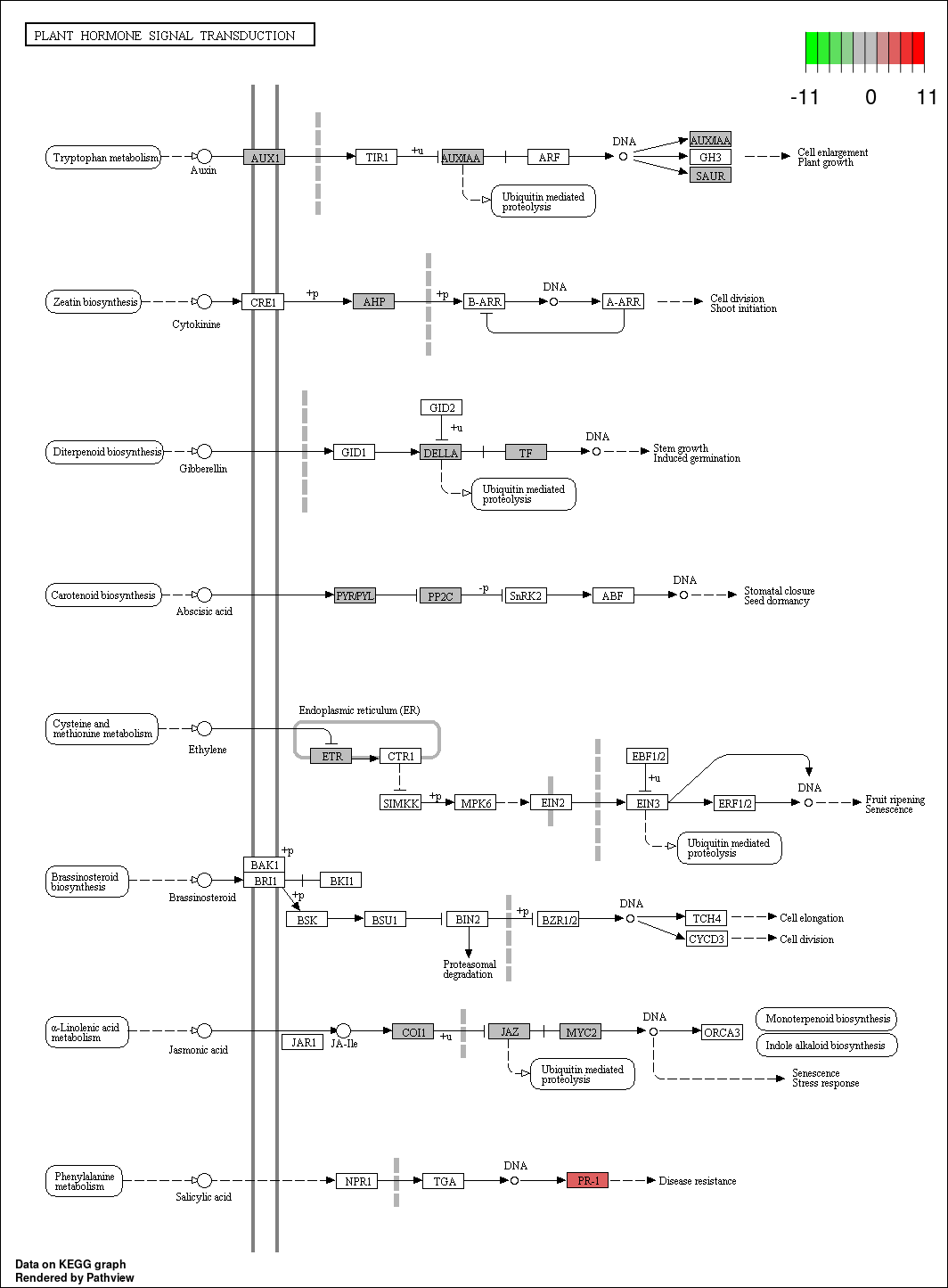

Supplement: Supplementary file 4 — Additional file 4: Fig. S4. Plant hormone signal transduction pathway in N1vs N0. [file 12870_2023_4263_MOESM4_ESM.zip › Figure S4.png]

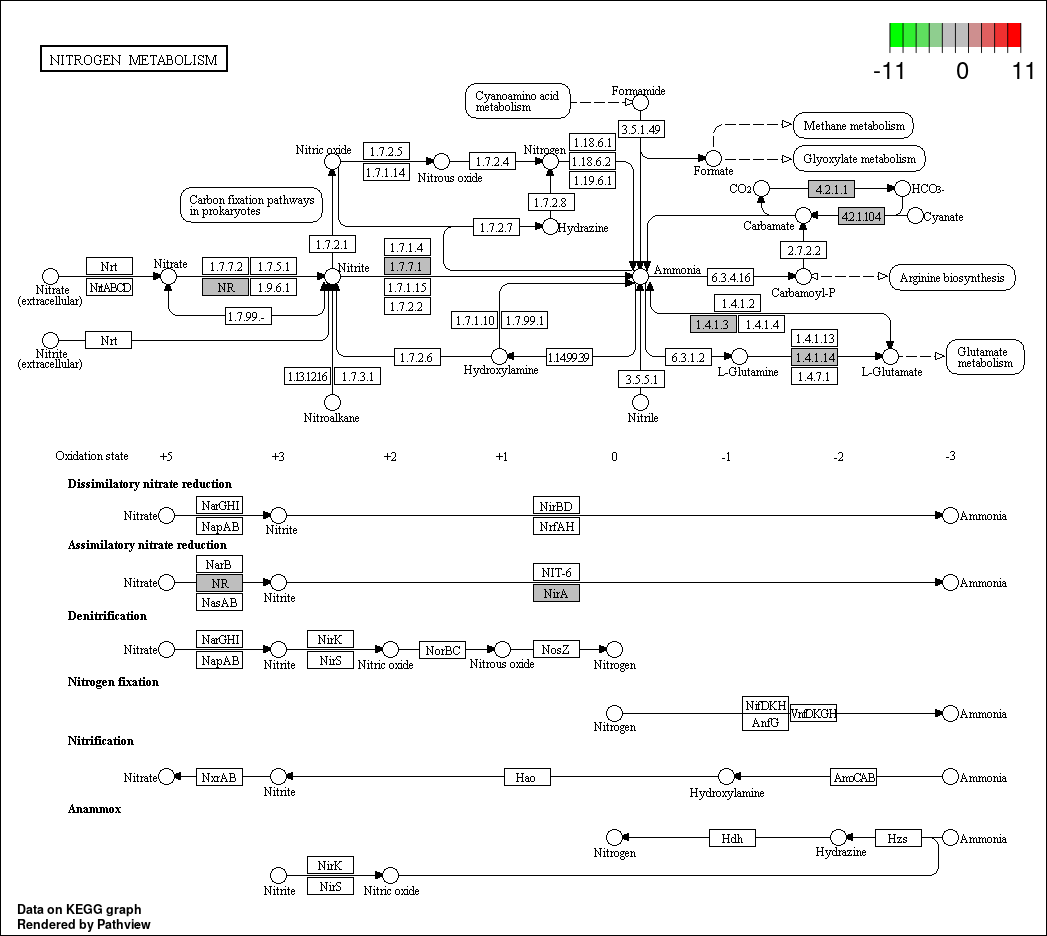

Supplement: Supplementary file 5 — Additional file 5: Fig. S5. Nitrogen metabolism pathway in N1vs N0. [file 12870_2023_4263_MOESM5_ESM.zip › Figure S5.png]

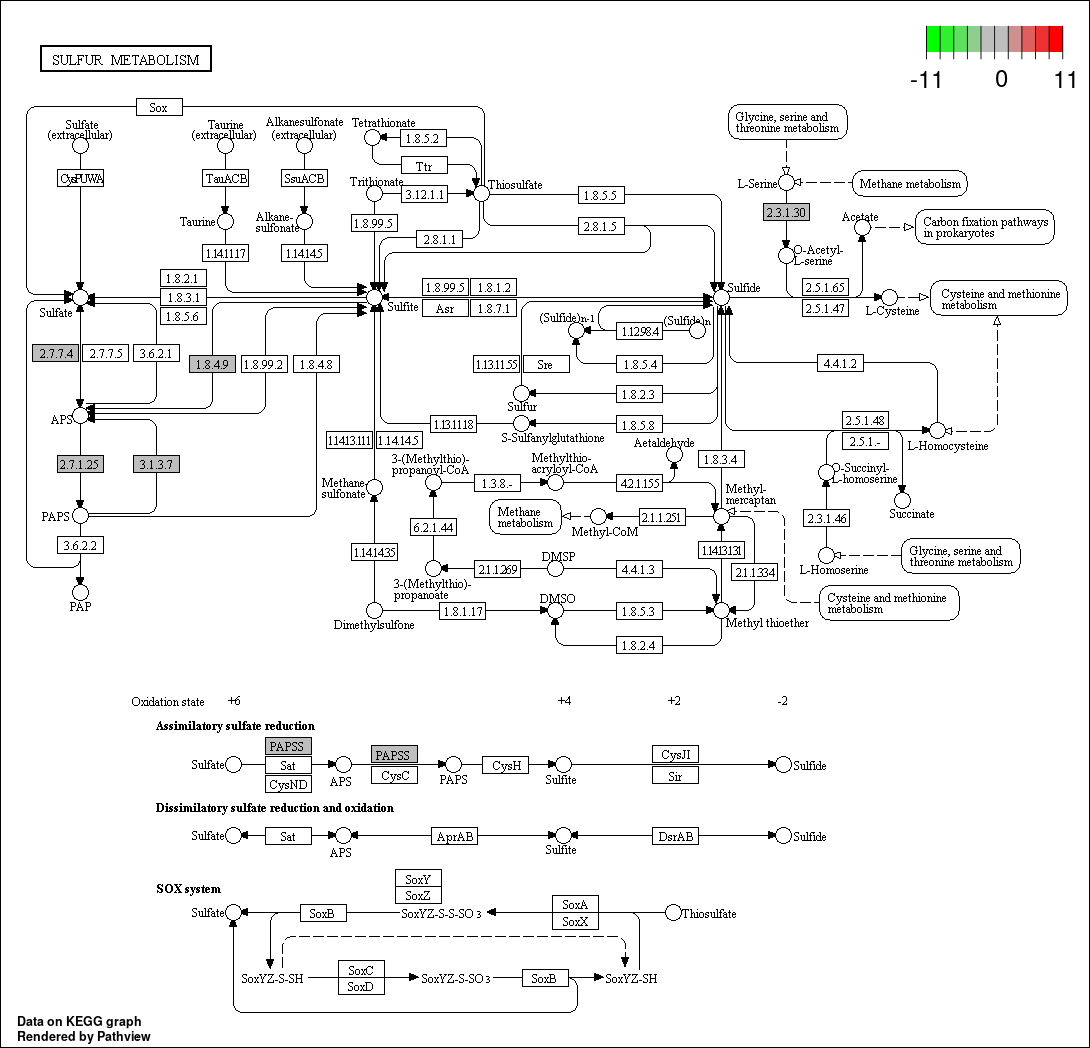

Supplement: Supplementary file 6 — Additional file 6: Fig. S6. Sulfur metabolism pathway in N1vs N0. [file 12870_2023_4263_MOESM6_ESM.zip › Figure S6.png]

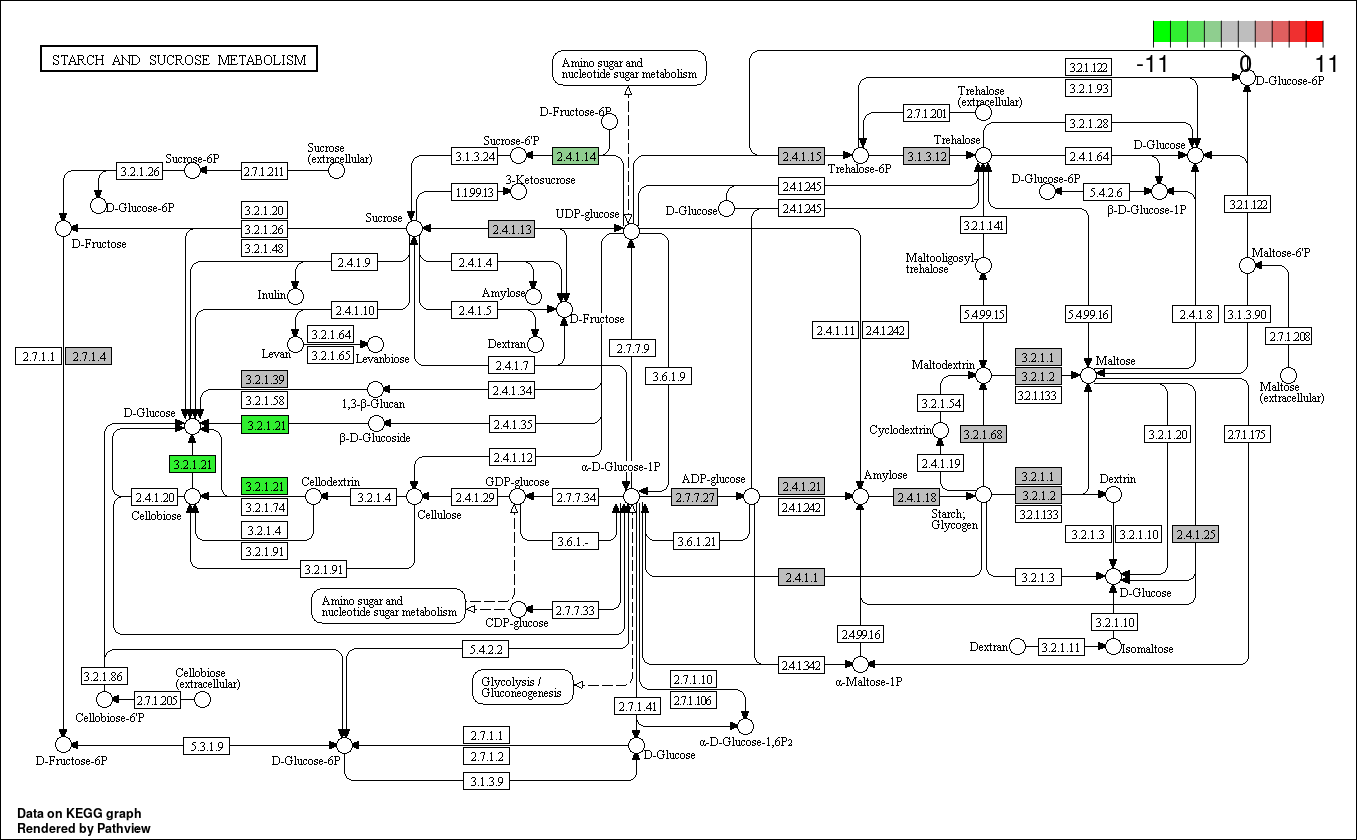

Supplement: Supplementary file 7 — Additional file 7: Fig. S7. Starch metabolism and sucrose pathway in N1vs N0. [file 12870_2023_4263_MOESM7_ESM.zip › Figure S7.png]

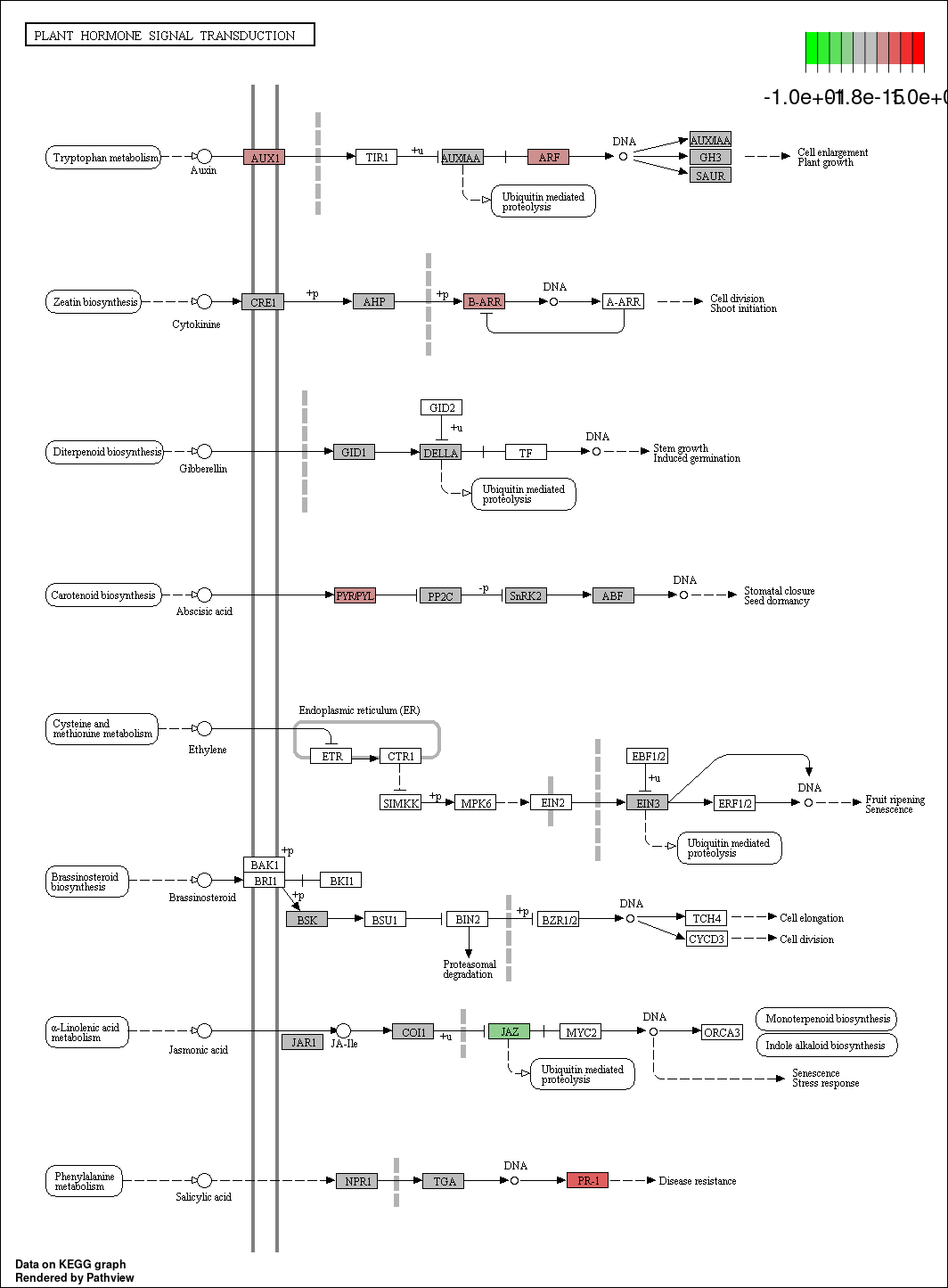

Supplement: Supplementary file 8 — Additional file 8: Fig. S8. Plant hormone signal transduction pathway in N2 vs N0. [file 12870_2023_4263_MOESM8_ESM.zip › Figure S8.png]

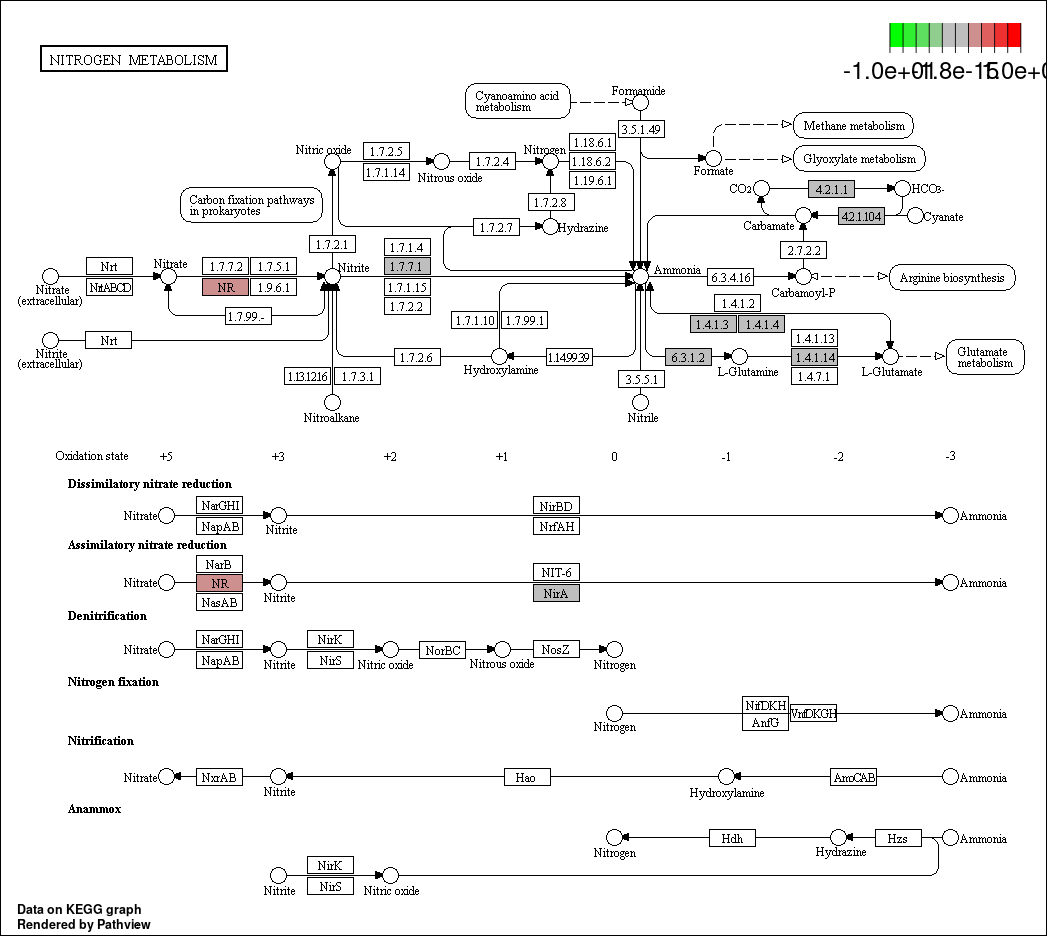

Supplement: Supplementary file 9 — Additional file 9: Fig. S9. Nitrogen metabolism pathway in N2 vs N0. [file 12870_2023_4263_MOESM9_ESM.zip › Figure S9.png]

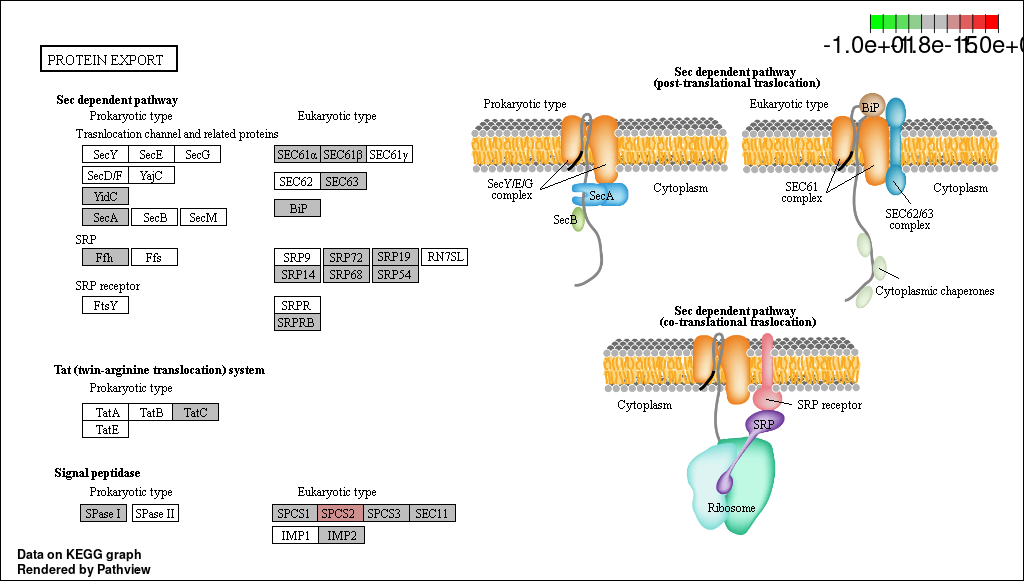

Supplement: Supplementary file 10 — Additional file 10: Fig. S10. Protein export pathway in N2 vs N0. [file 12870_2023_4263_MOESM10_ESM.zip › Figure S10.png]

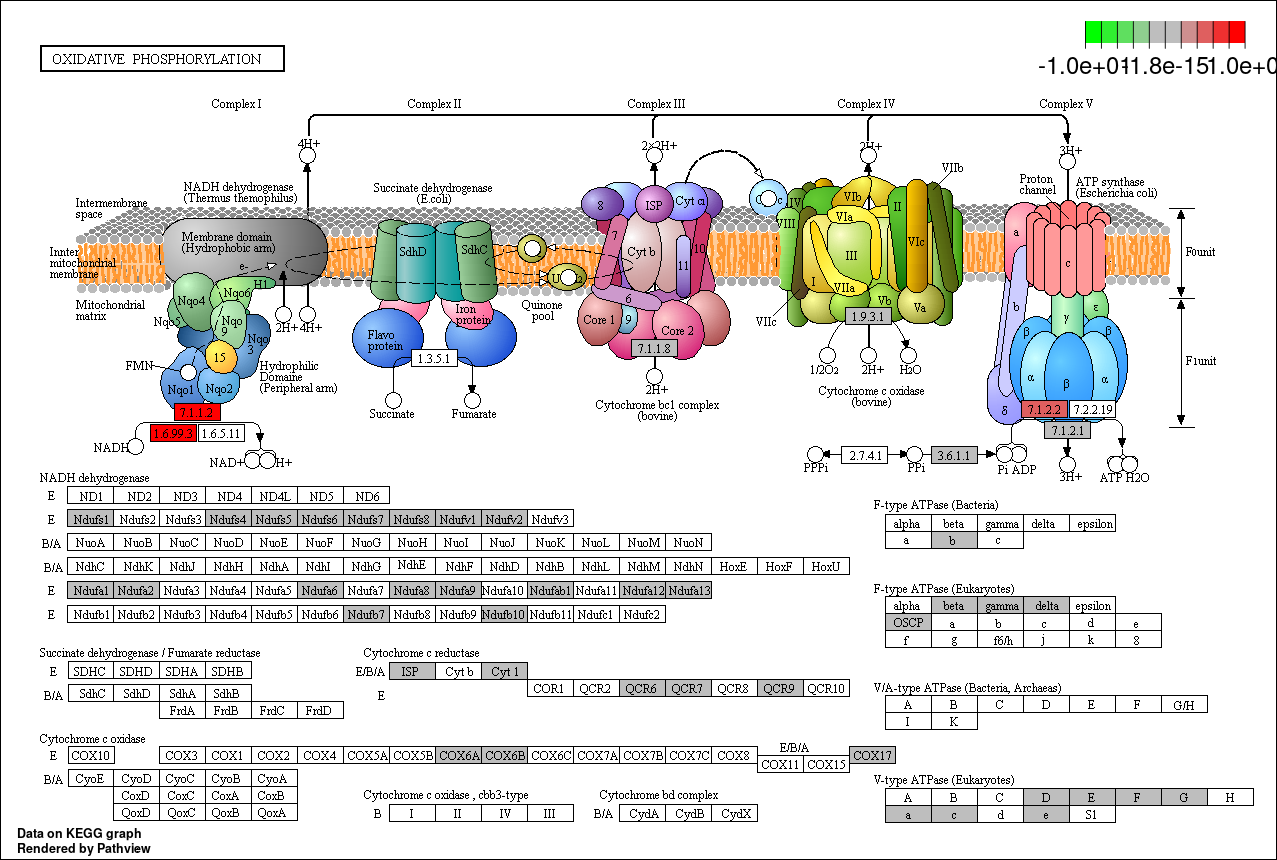

Supplement: Supplementary file 11 — Additional file 11: Fig. S11. Oxidative phospholylation pathway in N2 vs N0. [file 12870_2023_4263_MOESM11_ESM.zip › Figure S11.png]

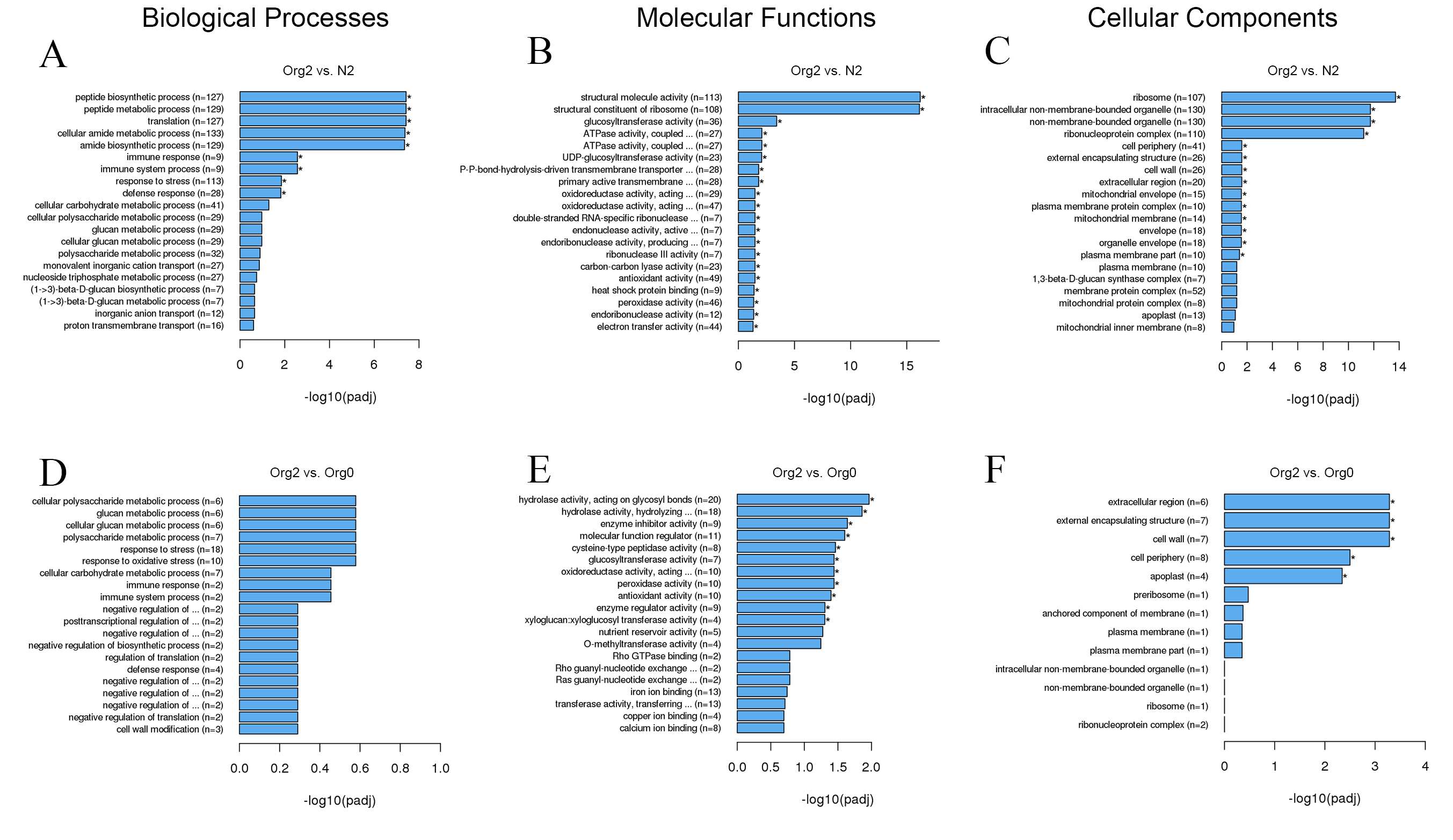

Supplement: Supplementary file 12 — Additional file 12: Fig. S12 GO Enrichment Histogram of top 20 enriched terms associated with DEGs of Org2 vs. N2 in biological processes (BP) (A), molecular functions (MF) (B), and cellular components (CC) (C); Org2 vs. Org0 in BP (D), MF (E), and CC (F). [file 12870_2023_4263_MOESM12_ESM.zip › Figure S12.tif]

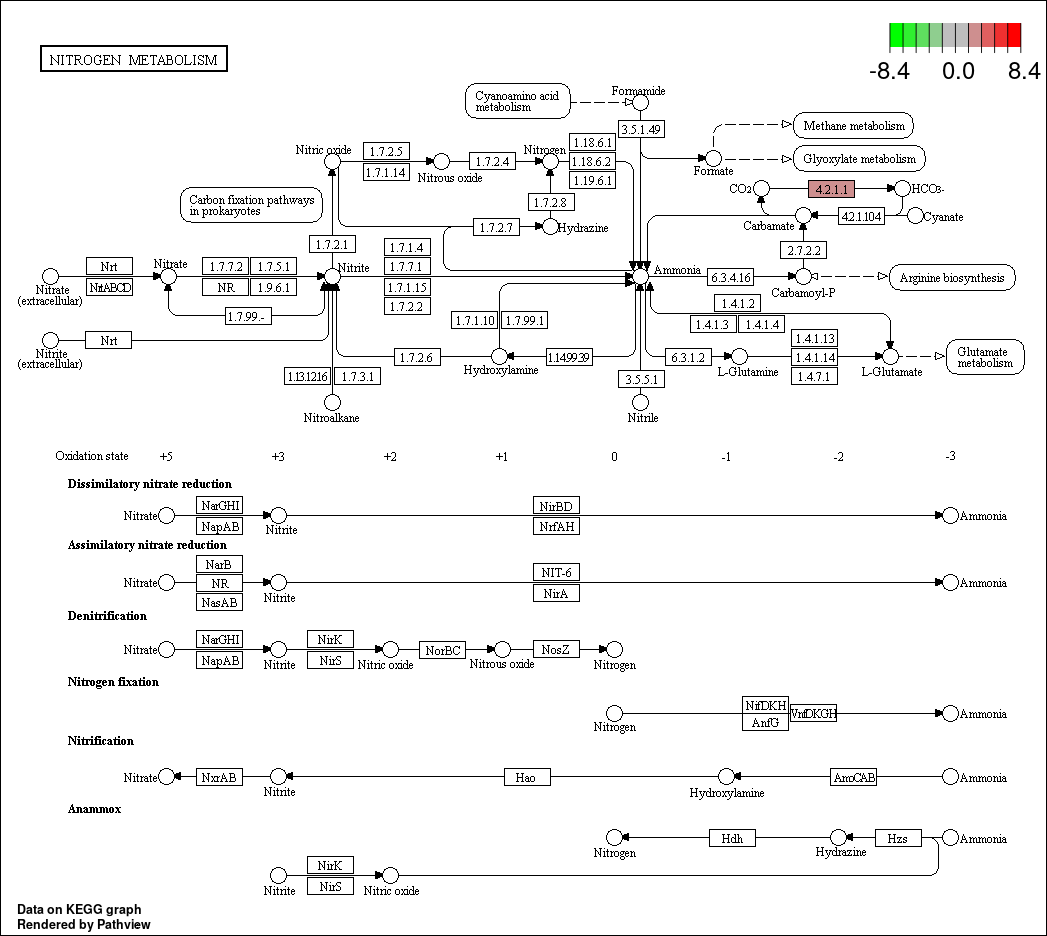

Supplement: Supplementary file 13 — Additional file 13: Fig. S13. Nitrogen metabolism pathway in Org2 vs Org0. [file 12870_2023_4263_MOESM13_ESM.zip › Figure S13.png]

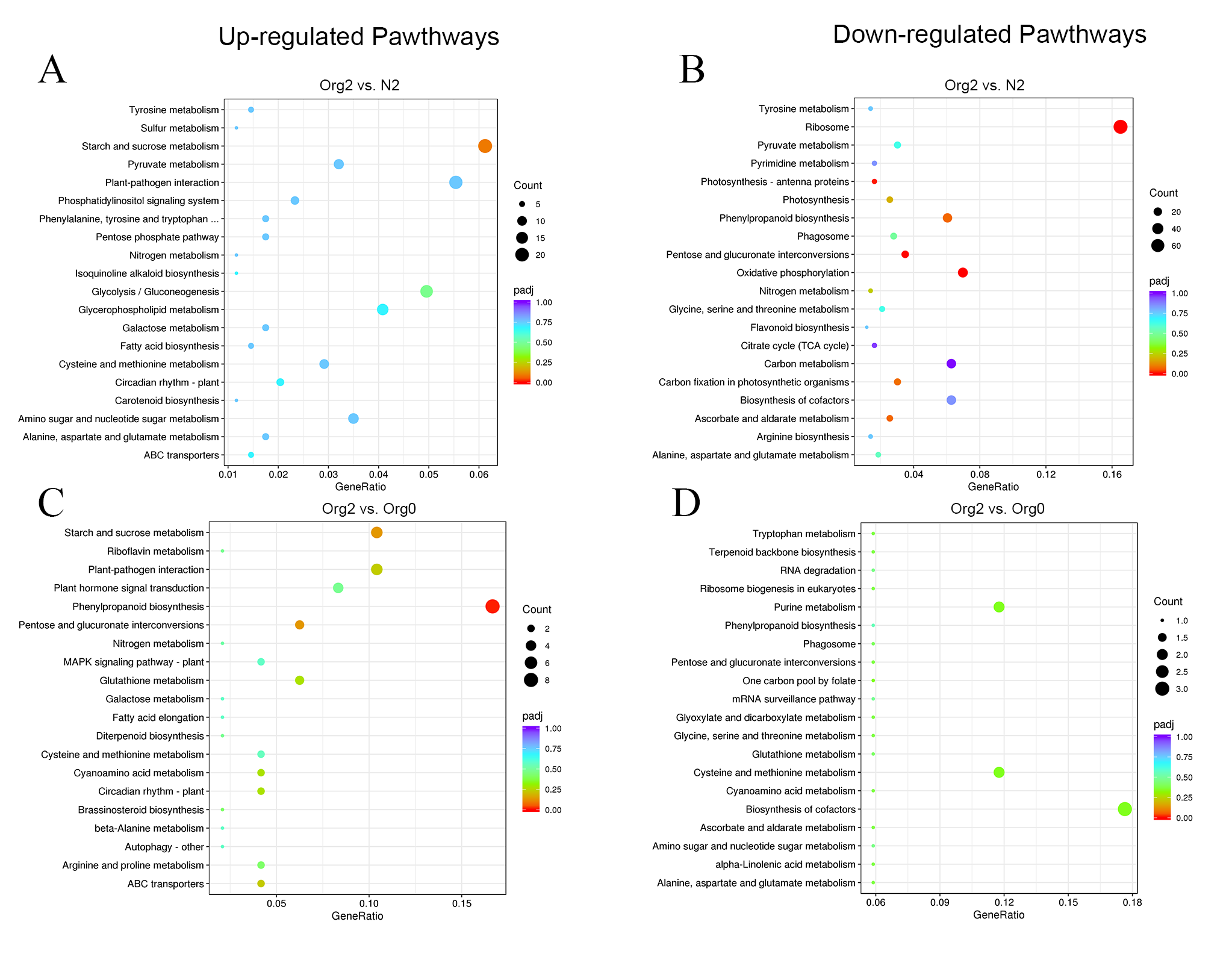

Supplement: Supplementary file 14 — Additional file 14: Fig. S14. KEGG Enrichment Scatter Plot of top 20 enriched terms associated with DEGs. Up- (A) and downregulated (B) pathways in Org2 vs. N2; up- (C) and downregulated (D) pathways in Org2 vs. Org0. [file 12870_2023_4263_MOESM14_ESM.zip › Figure S14.tif]

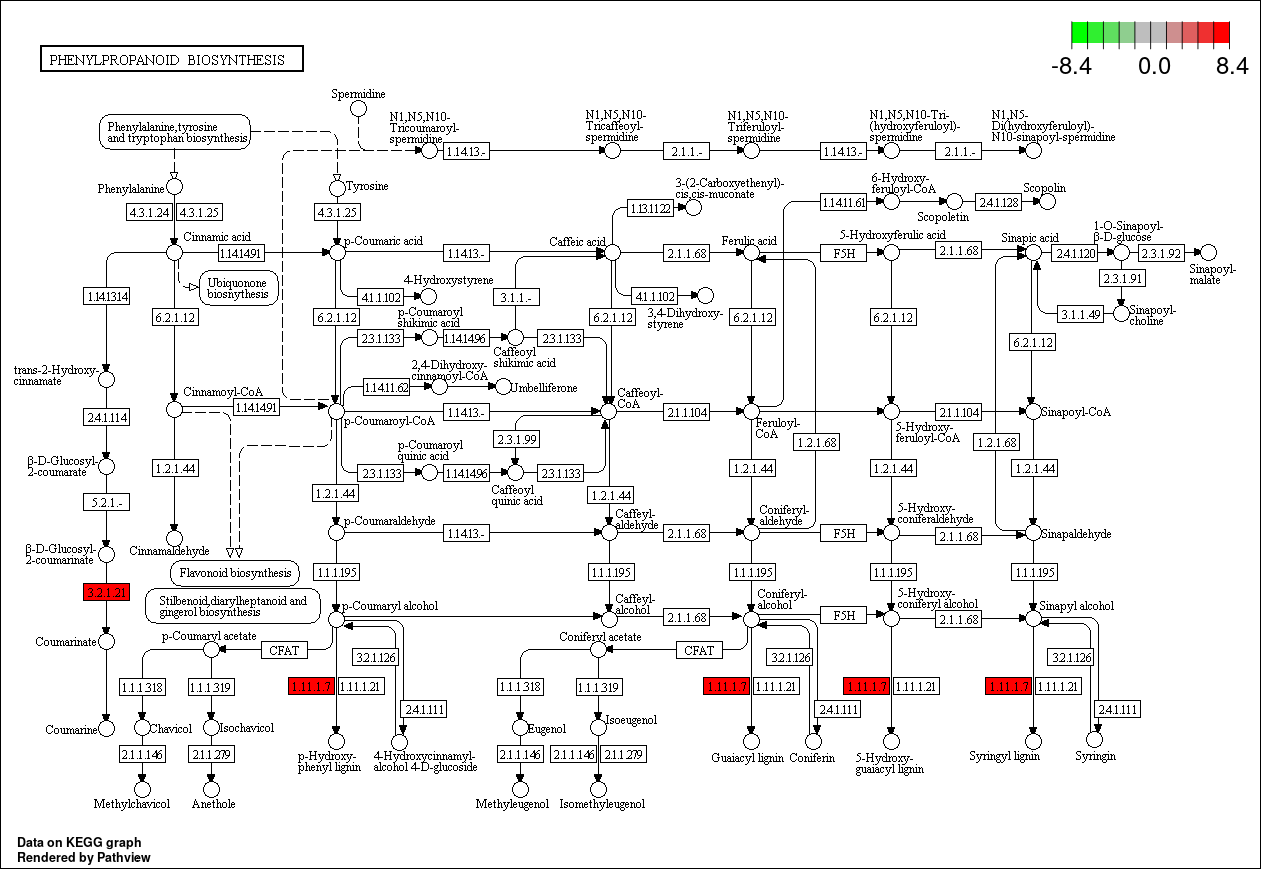

Supplement: Supplementary file 15 — Additional file 15: Fig. S15. Phenylpropanoid biosynthesis pathway in Org2 vs Org0. [file 12870_2023_4263_MOESM15_ESM.zip › Figure S15.png]

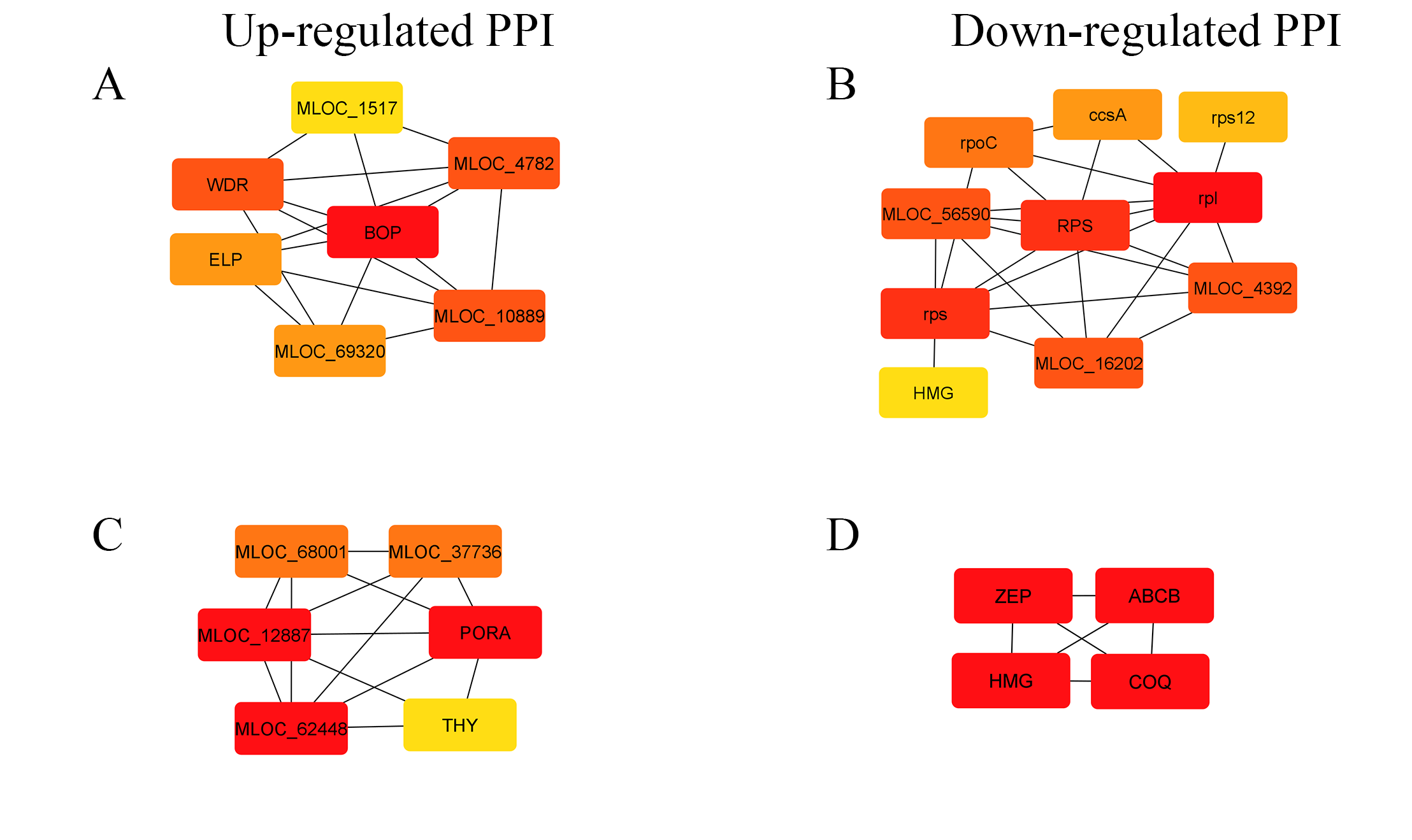

Supplement: Supplementary file 16 — Additional file 16: Fig. S16. PPI network of Up- (A) and downregulated (B) DEGs in Org2 vs. N2; PPI network of up- (C) and downregulated (D) DEGs in Org2 vs. Org0. [file 12870_2023_4263_MOESM16_ESM.zip › Figure S16.tif]

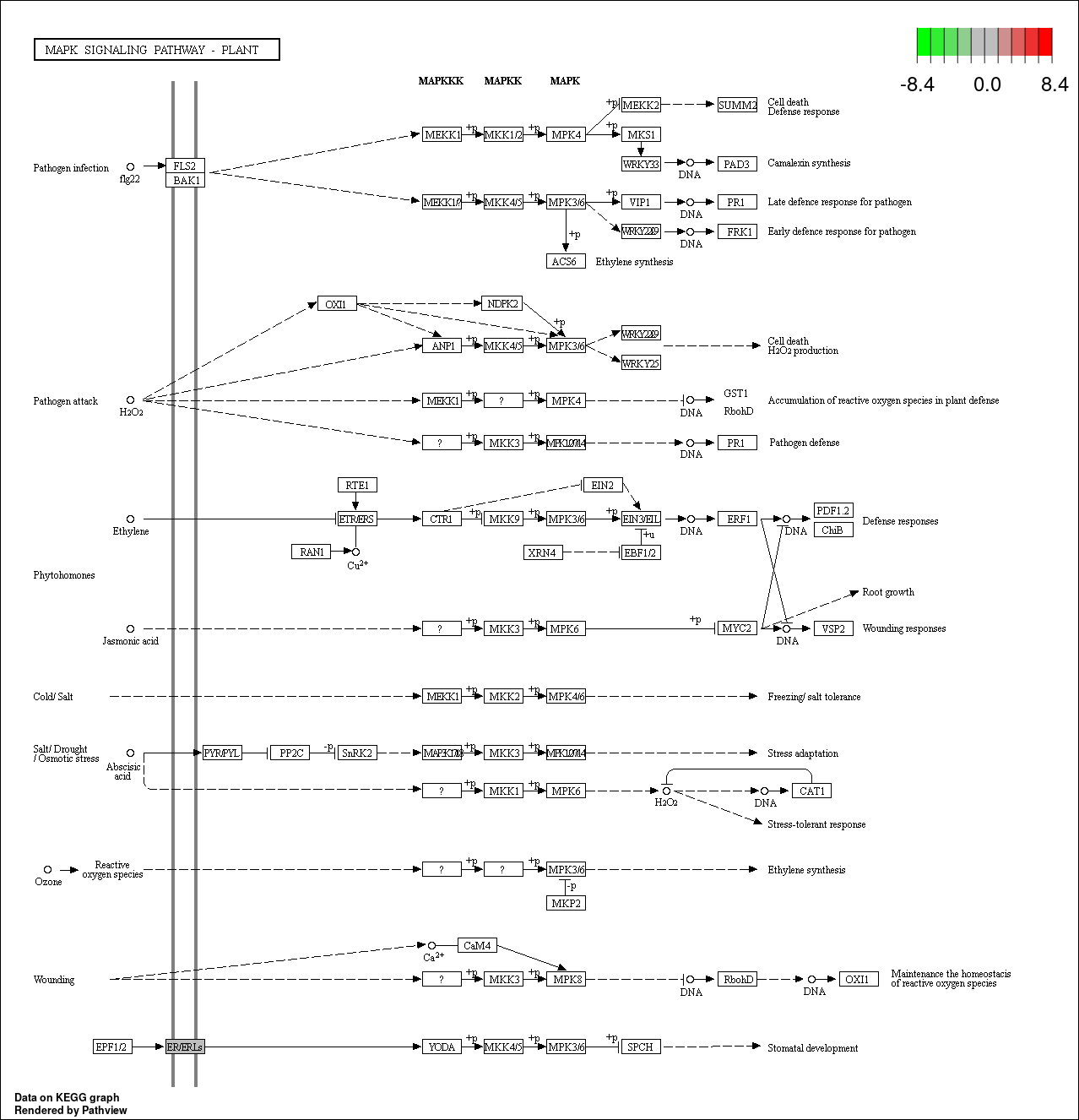

Supplement: Supplementary file 17 — Additional file 17: Fig. S17. MAPK signaling pathway in Org2 vs Org0. [file 12870_2023_4263_MOESM17_ESM.zip › Figure S17.png]

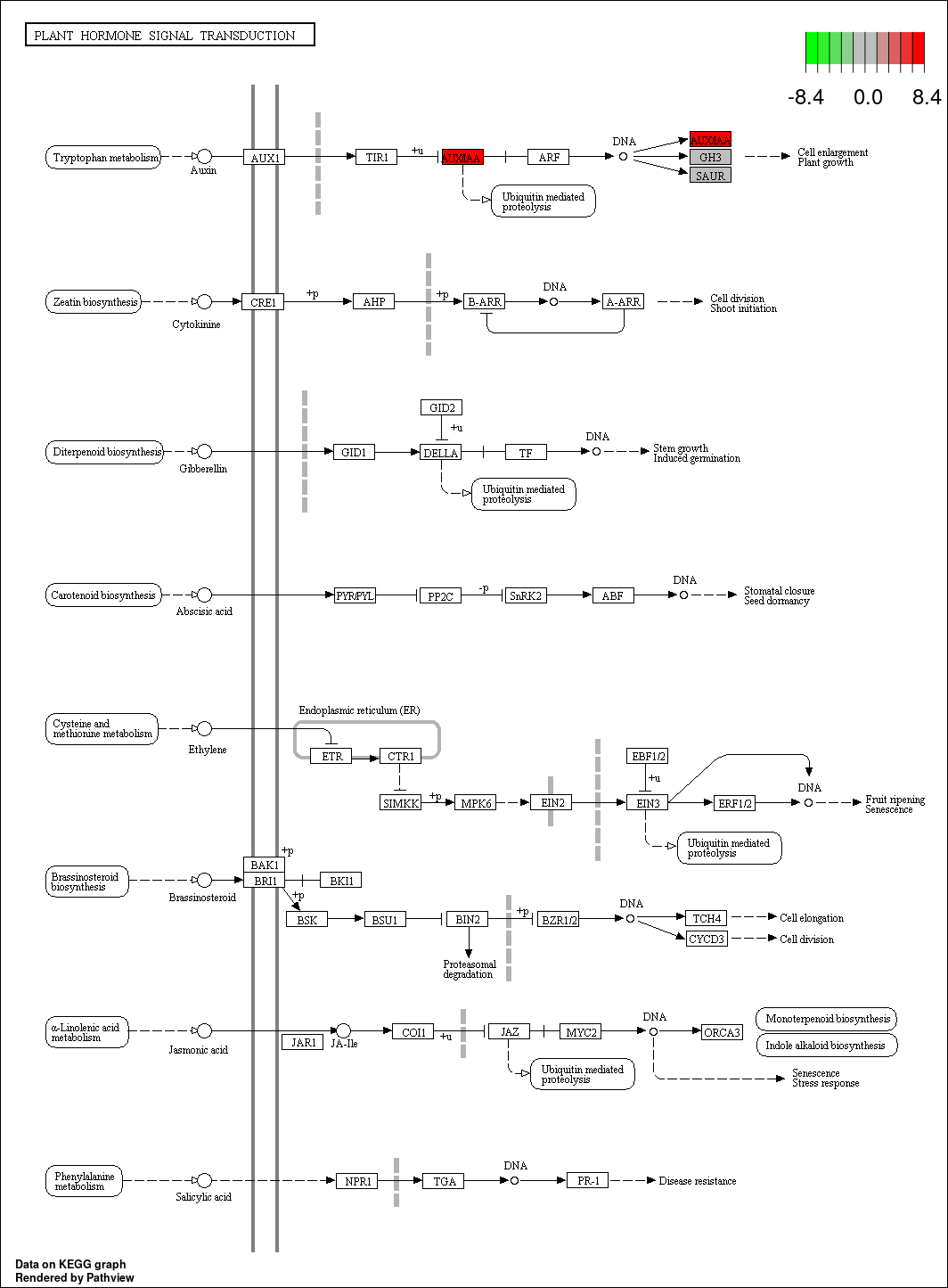

Supplement: Supplementary file 18 — Additional file 18: Fig. S18. Plant hormone signal transduction pathway in Org2 vs Org0. [file 12870_2023_4263_MOESM18_ESM.zip › Figure S18.png]

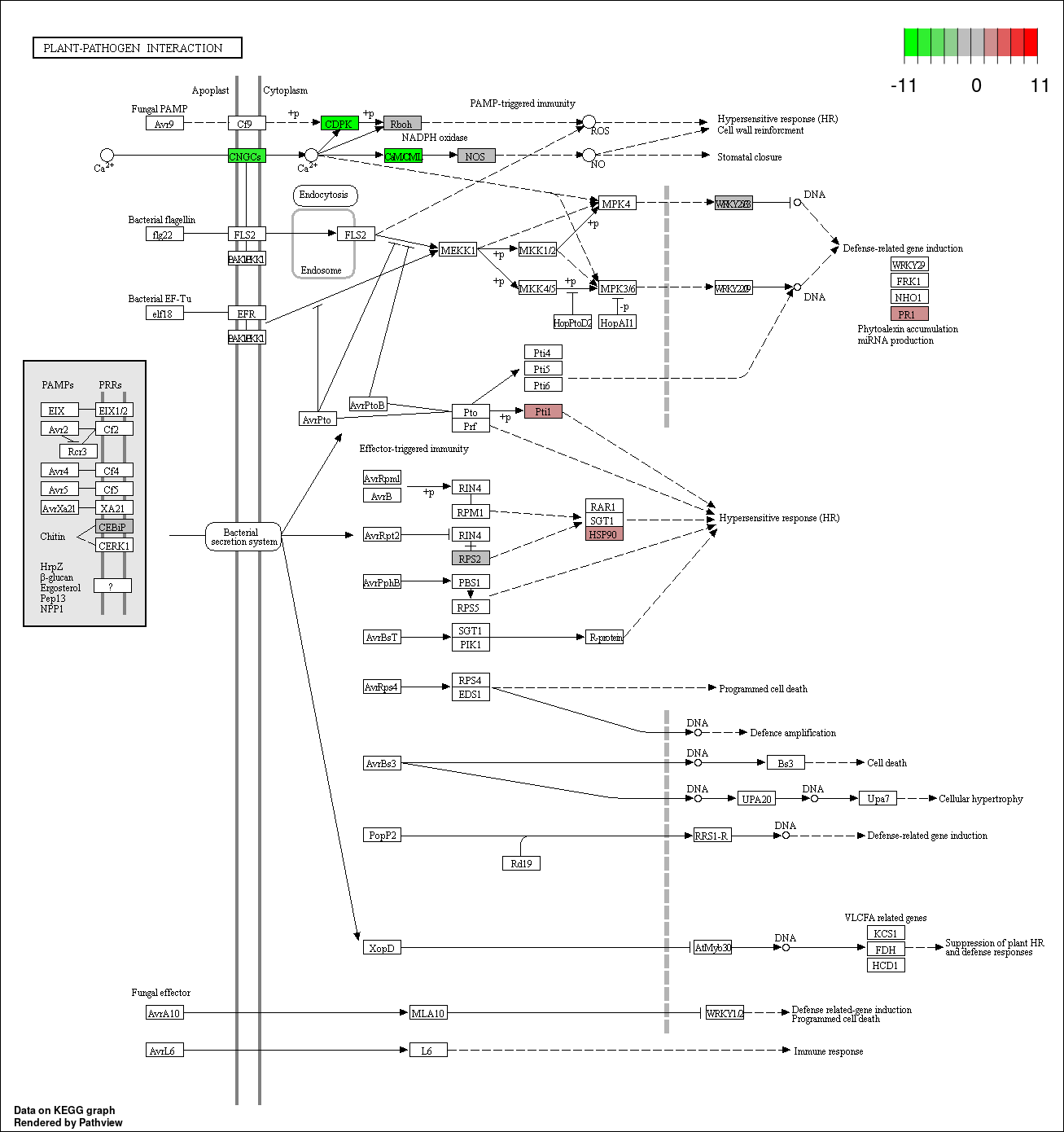

Supplement: Supplementary file 19 — Additional file 19: Fig. S19. Plant-pathogen interaction pathway in Org0 vs N0. [file 12870_2023_4263_MOESM19_ESM.zip › Figure S19.png]

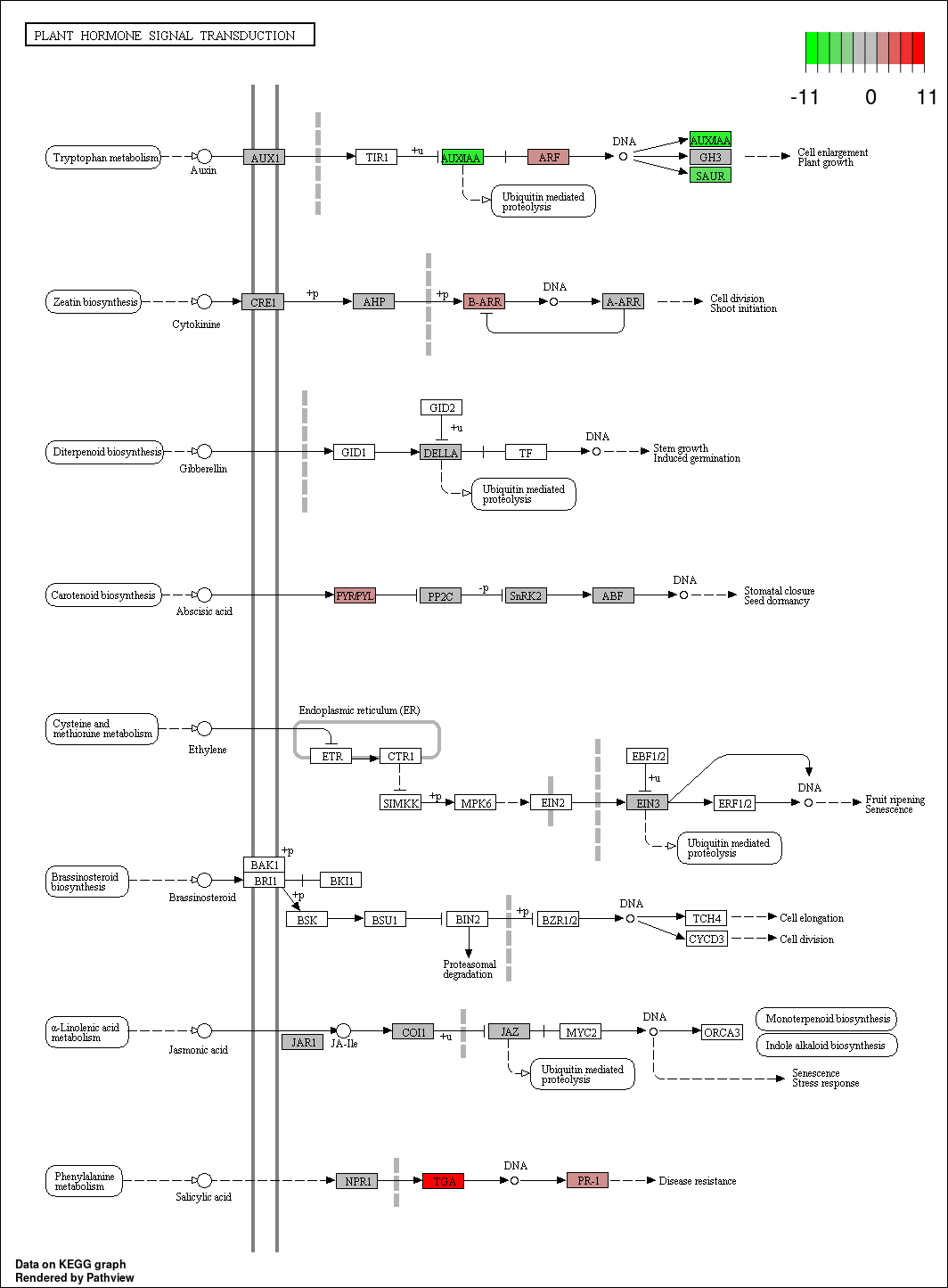

Supplement: Supplementary file 20 — Additional file 20: Fig. S20. Plant hormone signal transduction pathway in Org0 vs N0. [file 12870_2023_4263_MOESM20_ESM.zip › Figure S20.png]

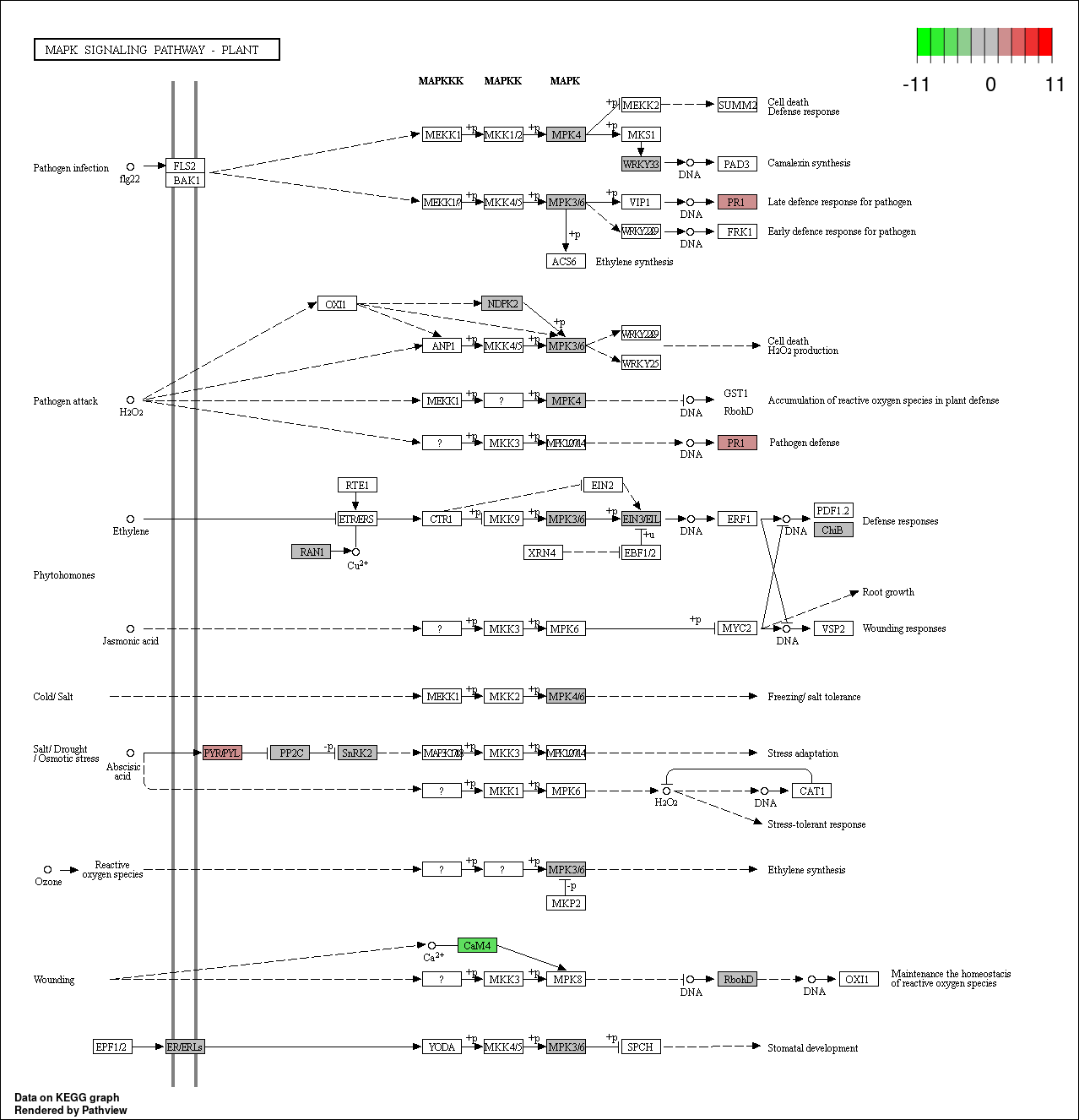

Supplement: Supplementary file 21 — Additional file 21: Fig. S21. MAPK signaling pathway in Org0 vs N0. [file 12870_2023_4263_MOESM21_ESM.zip › Figure S21.png]
