# Supplementary material for: Transcriptome profiling of barley in response to mineral and organic fertilizers
Source: BMC Plant Biol. 2023 May 16;23:261. doi: 10.1186/s12870-023-04263-2 (PMC10186687; doi:10.1186/s12870-023-04263-2)
Supplement: Supplementary file 2 — Additional file 2: Fig. S2. The statistics of whole differentially expressed genes. [file 12870_2023_4263_MOESM2_ESM.zip › Figure S2 caption.docx]

**Fig. S2** The statistics of whole differentially expressed genes.
