# Supplementary material for: Transcriptome profiling of barley in response to mineral and organic fertilizers
Source: BMC Plant Biol. 2023 May 16;23:261. doi: 10.1186/s12870-023-04263-2 (PMC10186687; doi:10.1186/s12870-023-04263-2)
Supplement: Supplementary file 12 — Additional file 12: Fig. S12 GO Enrichment Histogram of top 20 enriched terms associated with DEGs of Org2 vs. N2 in biological processes (BP) (A), molecular functions (MF) (B), and cellular components (CC) (C); Org2 vs. Org0 in BP (D), MF (E), and CC (F). [file 12870_2023_4263_MOESM12_ESM.zip › Figure S12 caption.docx]

**Fig. S12** GO Enrichment Histogram of top 20 enriched terms associated with DEGs of Org2 vs. N2 in biological processes (BP) (A), molecular functions (MF) (B), and cellular components (CC) (C); Org2 vs. Org0 in BP (D), MF (E), and CC (F).
