# Supplementary material for: Transcriptome profiling of barley in response to mineral and organic fertilizers
Source: BMC Plant Biol. 2023 May 16;23:261. doi: 10.1186/s12870-023-04263-2 (PMC10186687; doi:10.1186/s12870-023-04263-2)
Supplement: Supplementary file 14 — Additional file 14: Fig. S14. KEGG Enrichment Scatter Plot of top 20 enriched terms associated with DEGs. Up- (A) and downregulated (B) pathways in Org2 vs. N2; up- (C) and downregulated (D) pathways in Org2 vs. Org0. [file 12870_2023_4263_MOESM14_ESM.zip › Figure S14 caption.docx]

**Fig. S14** KEGG Enrichment Scatter Plot of top 20 enriched terms associated with DEGs. Up- (A) and downregulated (B) pathways in Org2 vs. N2; up- (C) and downregulated (D) pathways in Org2 vs. Org0.
