# Supplementary material for: Transcriptome profiling of barley in response to mineral and organic fertilizers
Source: BMC Plant Biol. 2023 May 16;23:261. doi: 10.1186/s12870-023-04263-2 (PMC10186687; doi:10.1186/s12870-023-04263-2)
Supplement: Supplementary file 16 — Additional file 16: Fig. S16. PPI network of Up- (A) and downregulated (B) DEGs in Org2 vs. N2; PPI network of up- (C) and downregulated (D) DEGs in Org2 vs. Org0. [file 12870_2023_4263_MOESM16_ESM.zip › Figure S16 caption.docx]

**Fig. S16** PPI network of Up- (A) and downregulated (B) DEGs in Org2 vs. N2; PPI network of up- (C) and downregulated (D) DEGs in Org2 vs. Org0.
