# Supplementary material for: Transcriptome profiling of barley in response to mineral and organic fertilizers
Source: BMC Plant Biol. 2023 May 16;23:261. doi: 10.1186/s12870-023-04263-2 (PMC10186687; doi:10.1186/s12870-023-04263-2)
Supplement: Supplementary file 17 — Additional file 17: Fig. S17. MAPK signaling pathway in Org2 vs Org0. [file 12870_2023_4263_MOESM17_ESM.zip › Figure S17 caption.docx]

**Fig S17** MAPK signaling pathway in Org2 vs Org0
