# Supplementary material for: Transcriptome profiling of barley in response to mineral and organic fertilizers
Source: BMC Plant Biol. 2023 May 16;23:261. doi: 10.1186/s12870-023-04263-2 (PMC10186687; doi:10.1186/s12870-023-04263-2)
Supplement: Supplementary file 20 — Additional file 20: Fig. S20. Plant hormone signal transduction pathway in Org0 vs N0. [file 12870_2023_4263_MOESM20_ESM.zip › Figure S20 caption.docx]

**Fig S20** Plant hormone signal transduction pathway in Org0 vs N0
