# Supplementary material for: Transcriptome profiling of barley in response to mineral and organic fertilizers
Source: BMC Plant Biol. 2023 May 16;23:261. doi: 10.1186/s12870-023-04263-2 (PMC10186687; doi:10.1186/s12870-023-04263-2)
Supplement: Supplementary file 30 — Additional file 30: Table S1. List of DEGs involved in the important KEGG pathways under different treatments. [file 12870_2023_4263_MOESM30_ESM.docx]

**Table S1** List of DEGs involved in the important KEGG pathways under different treatments

| KEGG term | DEGs |
| --- | --- |
| N1 vs N0 | |
| MAPK signaling pathway | mitogen-activated protein kinase 9-like, mitogen-activated protein kinase 4, probable ethylene response sensor 2 isoform X1, abscisic acid receptor PYL4, chitinase 8, chitinase 9, transcription factor MYC2, probable WRKY transcription factor 33, probable protein phosphatase 2C 30 |
| Plant hormone signal transduction pathway | auxin transporter-like protein 1, auxin-responsive protein SAUR36, auxin-responsive protein IAA19, auxin-responsive protein IAA31, auxin-induced protein 15A-like, abscisic acid receptor PYL4, probable ethylene response sensor 2 isoform X1, protein TIFY 11a, protein TIFY 11b, protein TIFY 11c, protein TIFY 6b, transcription factor PIF4 isoform X1, transcription factor MYC2, DELLA protein SLR1, coronatine-insensitive protein homolog 1b, coronatine-insensitive protein homolog 2, |
| Nitrogen metabolism | glutamate dehydrogenase, nitrate reductase [NADH] 1, glutamate synthase 1 [NADH]; chloroplastic isoform X1, ferredoxin--nitrite reductase; chloroplastic, alpha carbonic anhydrase 7 isoform X1, beta carbonic anhydrase 5; chloroplastic isoform X1, cyanate hydratase |
| Sulfur metabolism | probable serine acetyltransferase 5, ATP-sulfurylase 3; chloroplastic, probable 5'-adenylylsulfate reductase 1; chloroplastic, putative PAP-specific phosphatase; mitochondrial |
| Starch and sucrose metabolism | beta-glucosidase BoGH3B, beta-glucosidase 16 isoform X3, beta-glucosidase 30 isoform X1, probable inactive beta-glucosidase 33, beta-amylase 1; chloroplastic, beta-amylase 3; chloroplastic, endoglucanase 12, alpha-amylase 3; chloroplastic, alpha-amylase isozyme 2A, sucrose synthase 2, sucrose synthase 3, starch synthase 3; chloroplastic/amyloplastic, probable sucrose-phosphate synthase 5 isoform X2, probable sucrose-phosphate synthase 1, glucan endo-1,3-beta-glucosidase 6, isoamylase 2; chloroplastic, 1,4-alpha-glucan-branching enzyme 2; chloroplastic/amyloplastic, fructokinase-1 |
| Biosynthesis of amino acids | ribulose-phosphate 3-epimerase; cytoplasmic isoform, aminoacylase-1, tryptophan synthase alpha chain, citrate synthase 3; peroxisomal, aspartate aminotransferase; chloroplastic, arginase 1; mitochondrial, glutamate synthase 1 [NADH]; chloroplastic isoform X1, delta-1-pyrroline-5-carboxylate synthase, acetylornithine deacetylase, phosphoglycerate kinase; cytosolic, transaldolase, cysteine synthase-like, probable serine acetyltransferase 5, phosphoglycerate kinase, chloroplastic, pyruvate kinase; cytosolic isozyme, aminotransferase ALD1 homolog, 5-methyltetrahydropteroyltriglutamate-- homocysteine methyltransferase 1, pyruvate kinase isozyme G; chloroplastic isoform X1, ATP-dependent 6-phosphofructokinase 6, indole-3-glycerol phosphate synthase; chloroplastic, branched-chain-amino-acid aminotransferase 2; chloroplastic, 2,3-bisphosphoglycerate-independent phosphoglycerate mutase isoform X1, enolase |
| N2 vs N0 | |
| MAPK signaling pathway | mitogen-activated protein kinase 9-like, mitogen-activated protein kinase 4, mitogen-activated protein kinase 5 isoform X2, ETHYLENE INSENSITIVE 3-like 1 protein, abscisic acid receptor PYR1, abscisic acid receptor PYL2, abscisic acid receptor PYL4, chitinase 8, chitinase 9, probable WRKY transcription factor 33, serine/threonine-protein kinase SAPK3 |
| Plant hormone signal transduction pathway | auxin-responsive protein IAA1, auxin-responsive protein SAUR32, auxin transporter-like protein 1, auxin transporter-like protein 3, protein TIFY 6b, protein TIFY 10b, protein TIFY 10c, DELLA protein SLR1, ABSCISIC ACID-INSENSITIVE 5-like protein 7, abscisic acid receptor PYR1, abscisic acid receptor PYL2, abscisic acid receptor PYL4, jasmonic acid-amido synthetase JAR2, serine/threonine-protein kinase SAPK3, coronatine-insensitive protein homolog 1b, coronatine-insensitive protein homolog 2 |
| Nitrogen metabolism | glutamate dehydrogenase, nitrate reductase [NADH] 1, ferredoxin--nitrite reductase; chloroplastic, glutamine synthetase cytosolic isozyme 1-1, glutamine synthetase cytosolic isozyme 1-2, glutamate synthase 1 [NADH]; chloroplastic isoform X1, cyanate hydratase, beta carbonic anhydrase 5; chloroplastic isoform X1, NADP-specific glutamate dehydrogenase isoform X1 |
| Oxidative phosphorylation | soluble inorganic pyrophosphatase, NADH dehydrogenase [ubiquinone] iron-sulfur protein 1; mitochondrial, NADH dehydrogenase [ubiquinone] iron-sulfur protein 4; mitochondrial, NADH dehydrogenase [ubiquinone] flavoprotein 1; mitochondrial, NADH dehydrogenase [ubiquinone] 1 alpha subcomplex subunit 2,  NADH dehydrogenase [ubiquinone] 1 alpha subcomplex subunit 6, NADH dehydrogenase [ubiquinone] 1 beta subcomplex subunit 7, NADH-ubiquinone oxidoreductase subunit 8, cytochrome c oxidase subunit 6a, cytochrome c oxidase subunit 6b-1, cytochrome c oxidase copper chaperone 1, cytochrome b-c1 complex subunit 6, cytochrome c1-2; heme protein; mitochondrial, ATP synthase subunit beta; mitochondrial-like, ATP synthase subunit delta; mitochondrial, ATP synthase subunit gamma; mitochondrial, V-type proton ATPase 16 kDa proteolipid subunit |
| Protein export | protein transport protein Sec61 subunit alpha, protein transport protein Sec61 subunit beta, signal recognition particle 54 kDa protein 2, signal recognition particle 19 kDa protein, signal recognition particle 14 kDa protein isoform X1, signal recognition particle receptor subunit beta, signal peptidase complex catalytic subunit SEC11A, protein translocase subunit SECA1; chloroplastic  protein translocase subunit SECA2; chloroplastic isoform X1, signal peptidase complex subunit 3B, signal peptidase complex subunit 1, mitochondrial inner membrane protein OXA1-like, mitochondrial inner membrane protease subunit 2, luminal-binding protein 3, dnaJ protein ERDJ2A |
| Biosynthesis of amino acids | ribulose-phosphate 3-epimerase; cytoplasmic isoform, aspartate aminotransferase; chloroplastic, argininosuccinate synthase; chloroplastic, aspartate aminotransferase; chloroplastic, citrate synthase 3; peroxisomal,  aminoacylase-1, arginase 1; mitochondrial  delta-1-pyrroline-5-carboxylate synthase, phosphoglycerate kinase; cytosolic, pyruvate kinase isozyme A; chloroplastic, ATP-dependent 6-phosphofructokinase 6, branched-chain-amino-acid aminotransferase 2; chloroplastic, enolase, glyceraldehyde-3-phosphate dehydrogenase 3; cytosolic, probable serine acetyltransferase 2, serine hydroxymethyltransferase 4, glyceraldehyde-3-phosphate dehydrogenase 1; cytosolic, triosephosphate isomerase; cytosolic, glutamine synthetase cytosolic isozyme 1-1  argininosuccinate synthase; chloroplastic, 2-isopropylmalate synthase A, 3-isopropylmalate dehydratase small subunit 3, isocitrate dehydrogenase [NADP], transaldolase, asparagine synthetase [glutamine-hydrolyzing] 1, acetolactate synthase 1; chloroplastic, aspartate-semialdehyde dehydrogenase |
| Org0 vs N0 | |
| MAPK signaling pathway | pathogenesis-related protein PRB1-3, mitogen-activated protein kinase 2, mitogen-activated protein kinase 5 isoform X2, ETHYLENE INSENSITIVE 3-like 1 protein, abscisic acid receptor PYL2, probable WRKY transcription factor 33, calmodulin-3, chitinase 2, chitinase 11, serine/threonine-protein kinase SAPK3, serine/threonine-protein kinase SAPK5, serine/threonine-protein kinase SAPK7, LRR receptor-like serine/threonine-protein kinase ERECTA, LRR receptor-like serine/threonine-protein kinase ERL1, nucleoside diphosphate kinase 3 |
| Plant hormone signal transduction pathway | auxin-responsive protein IAA1, auxin-responsive protein IAA14, auxin-responsive protein IAA19, auxin-responsive protein SAUR36, auxin-induced protein 15A-like, auxin transporter-like protein 1, transcription factor HBP-1b(c1), transcription factor HBP-1b(c38) isoform X1, transcription factor TGA2 isoform X2, abscisic acid receptor PYL2, ABSCISIC ACID-INSENSITIVE 5-like protein 7, jasmonic acid-amido synthetase JAR1, ETHYLENE INSENSITIVE 3-like 1 protein, protein TIFY 6b, protein TIFY 10c, regulatory protein NPR6, DELLA protein SLN1, pathogenesis-related protein PRB1-3, serine/threonine-protein kinase SAPK7, serine/threonine-protein kinase SAPK3 |
| Nitrogen metabolism | alpha carbonic anhydrase 7, beta carbonic anhydrase 5, glutamate dehydrogenase, ferredoxin-dependent glutamate synthase; chloroplastic, glutamine synthetase cytosolic isozyme 1-2 |
| Plant-pathogen interaction | heat shock protein 90, heat shock protein 82, respiratory burst oxidase homolog protein B, disease resistance protein RPM1 isoform X1, disease resistance protein RPS2, pathogenesis-related protein PRB1-3, probable WRKY transcription factor 33, calcium-dependent protein kinase 7, probable calcium-binding protein CML12, PTI1-like tyrosine-protein kinase 1, mitogen-activated protein kinase 2, chitin elicitor-binding protein |
| Org2 vs Org0 | |
| MAPK signaling pathway | LRR receptor-like serine/threonine-protein kinase ERECTA, LRR receptor-like serine/threonine-protein kinase ERECTA isoform X1 |
| Plant hormone signal transduction pathway | auxin-responsive protein SAUR36, auxin-responsive protein IAA27, auxin-responsive protein IAA14, probable indole-3-acetic acid-amido synthetase GH3.4, jasmonic acid-amido synthetase JAR2, |
| Nitrogen metabolism | alpha carbonic anhydrase 7 isoform X1 |
| Phenylpropanoid biosynthesis | beta-glucosidase BoGH3B, beta-glucosidase 1, peroxidase 40, peroxidase 5, peroxidase 72, peroxidase 21, peroxidase P7 |
| Starch and sucrose metabolism | beta-glucosidase BoGH3B, beta-glucosidase 1, endoglucanase 8, endoglucanase 6 |
| ABC transporters | ABC transporter B family member 9, ABC transporter B family member 11 |
